# Abundant Oligonucleotides Common to Most Bacteria

**DOI:** 10.1371/journal.pone.0009841

**Published:** 2010-03-23

**Authors:** Colin F. Davenport, Burkhard Tümmler

**Affiliations:** Pediatric Pneumology and Neonatology, Hanover Medical School, Hanover, Lower Saxony, Germany; University of Stellenbosch, South Africa

## Abstract

**Background:**

Bacteria show a bias in their genomic oligonucleotide composition far beyond that dictated by G+C content. Patterns of over- and underrepresented oligonucleotides carry a phylogenetic signal and are thus diagnostic for individual species. Patterns of short oligomers have been investigated by multiple groups in large numbers of bacteria genomes. However, global distributions of the most highly overrepresented mid-sized oligomers have not been assessed across all prokaryotes to date. We surveyed overrepresented mid-length oligomers across all prokaryotes and normalised for base composition and embedded oligomers using zero and second order Markov models.

**Principal Findings:**

Here we report a presumably ancient set of oligomers conserved and overrepresented in nearly all branches of prokaryotic life, including Archaea. These oligomers are either adenine rich homopurines with one to three guanine nucleosides, or homopyridimines with one to four cytosine nucleosides. They do not show a consistent preference for coding or non-coding regions or aggregate in any coding frame, implying a role in DNA structure and as polypeptide binding sites. Structural parameters indicate these oligonucleotides to be an extreme and rigid form of B-DNA prone to forming triple stranded helices under common physiological conditions. Moreover, the narrow minor grooves of these structures are recognised by DNA binding and nucleoid associated proteins such as HU.

**Conclusion:**

Homopurine and homopyrimidine oligomers exhibit distinct and unusual structural features and are present at high copy number in nearly all prokaryotic lineages. This fact suggests a non-neutral role of these oligonucleotides for bacterial genome organization that has been maintained throughout evolution.

## Introduction

Bacterial genomes may vary widely in nucleotide content. This is most readily observable in region specific G+C content [1]. However, higher order oligonucleotide composition fluctuates far more within a genome than simple G+C content would suggest [2], [3]. This composition may be maintained due to replication and repair machinery, restriction modification or DNA structural constraints [4], [5]. As such, oligonucleotide biases represent an additional source of information which can be used to characterise a genome. For example, patterns of over- and underrepresented oligonucleotides carry a phylogenetic signal and are thus diagnostic for many individual species [2], [3], [6], [7].

Shorter oligomers up to octamers have now been exhaustively investigated by various groups. Karlin and colleagues produced a series of papers mainly focussing on dinucleotide usage and its application in genome analysis [4]. Dinucleotide compositions were also used to demonstrate that the genomic signature of plasmids is different from the host chromosomes with which they are associated [8]. Chaos game methods and their visualisations were also shown to characterise genomic composition and relatedness of organisms based on oligomer usage using 1 to 8mers in the genomes available at that time [7]. The evolutionary signal of tetranucleotides was analysed using Markov Chain models across multiple species and a broad similarity to 16S ribosomal RNA based trees was noted [5]. Other workers surveyed the information content of short oligomers across the prokaryotes and found hexamers to be optimal [9].

Longer oligomers of eight or more bp have also been investigated in restricted single or small groups of genomes. Karlin and coworkers looked at mid-length oligomers in *Haemophilus influenzae* [10] and three streptococci [11]. The same authors later looked at frequent medium-sized oligomers of 8–11 bp in large viral genomes [12]. Oligomers in yeast were surveyed by Hampson and colleagues [13]. The organisation of mitochondrial genomes were examined using chaos game representations by Wang and coworkers [14]. Chor and colleagues investigated the entire oligomer spectrum of over a hundred prokaryotic and eukaryotic genomes, but concentrated on modalities of the distributions rather than highly frequent words [15]. In summary, efforts to characterise usage of mid-length oligomers have been targetted towards specific taxonomic groups, but to our knowledge no comprehensive analysis of the prokaryotes has been undertaken.

We here report oligonucleotides overrepresented across 684 sequenced chromosomes from diverse lineages of the prokaryotic world. These related and complementary oligonucleotides are characterised by the presence of A-tracts, runs of adenines which do not contain the flexible A-T step [16]. Structural parameters indicate these oligomers to be bent and highly propellor twisted, with a narrow minor groove. We suggest these oligomers play a role, consistent with past observations in prokaryotes and analogous to nucleosome association in eukaryotes, as binding sites for enzymes responsible for packaging of the bacterial nucleoid.

## Materials and Methods

The program OligoCounter [3], (available at http://webhost1.mh-hannover.de/davenport/oligocounter/), was used to to count overrepresented 8–14 bp oligomers in the whole genomes of 684 chromosomes available from the NCBI FTP site (February 2008).

### Initial dataset

**Figure 1:**
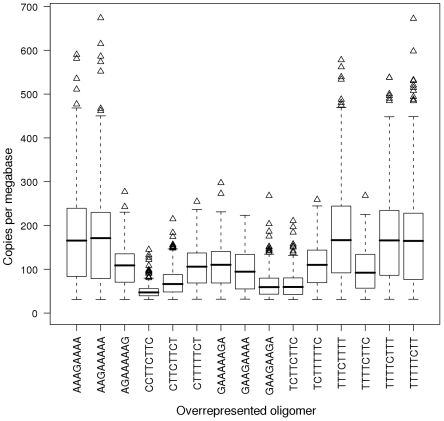
Normalised copy numbers of each oligomer. Box and whisker plots showing the distribution of copy numbers per megabase for the 15 overrepresented oligomers for all chromosomes in which they were overrepresented. The upper end of the dashed line is the 95% confidence interval, beyond which outlier chromosomes with very high copy numbers are depicted as triangles. The lower limit is set by the lower threshold of 31 oligomer copies per megabase, i.e. twice the expected value of 15.2 for a randomly distributed octamer in one megabase. Note that GC content was previously controlled for by the zero-order and second-order Markov models used to select and verify the datasets respectively.

OligoCounter thresholds were set to retain oligomers present at least 31 times per Mb in the genome with a χ^2^- value of 100 or more. We estimate 15.2 ( = 10^6^/4^8^) copies of a random octamer are expected to be present in each Mb of a prokaryotic genome. Thus, a threshold of twice this value (31) together with the χ^2^ cut-off restricted the oligomers we analysed. It should however be noted that median values of the located oligomers were far in excess of this value (Figure 1). χ^2^-statistics were calculated according to the following formula [17]:
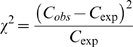
where C_obs_ is the observed count of words and C_exp_ is the expected count of words.

Expected counts of oligomers for the initial dataset were derived by a zero-order Markov model, which controls for genome size and mononucleotide content [18], while later selections were based on a second-order Markov model (see below):
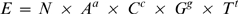
where *N* is the genome size in nucleotides, *A* is the proportion of adenine in the genome and *a* is the number of adenines in the oligo, and so on for the other bases. The χ^2^- statistic is here not used as an indicator for statistical significance but merely of level of overrepresentation of each oligomer, otherwise Bonferroni corrections for multiple tests would have been necessary. A random 6 Mb genome with 50% G+C content was generated as a control and demonstrated to have no oligonucleotide biases at a χ^2^-value of 100.

### Oligomer selection strategy

We compared all oligomers from five strains belonging to a selection of the most phylogenetically distinct lineages (Spirochaetes, Chlamydiae, Bacteroidetes/Chlorobi and Cyanobacteria, and either Firmicutes or Gammaproteobacteria). Our hypothesis was that oligomers common to all of these taxa would also be found in many others. This process was performed with four sets of strains of differing G+C content to confirm the robustness of this hypothesis.

**Set 1** (Average G+C  = 37%): *Clostridium tetani* (NC_004557), *Leptospira interrogans serovar Copenhageni* (NC_005823), *Chlamydia trachomatis* A/HAR-13 (NC_007429), *Bacteroides fragilis* NCTC 9343 (NC_003228), *Prochlorococcus marinus* str. MIT 9211 (NC_009976).

**Set 2** (Average G+C  = 50.8%): *Hahella chejuensis* (NC_007645), *Porphyromonas gingivalis* W83 (NC_002950), *Chlamydophila pneumoniae* AR39 (NC_002179), *Treponema pallidum* subsp. *pallidum* str. Nichols (NC_000919), *Gloeobacter violaceus* PCC 7421 (NC_005125).

**Set 3** (Average G+C  = 37.8%): *Streptococcus pneumoniae* R6 (NC_003098), *Borrelia afzelii* (NC_008277), *Chlamydophila felis* Fe-C-56 (NC_007899), *Bacteroides thetaiotaomicron* VPI-5482 (NC_004663), *Anabaena variabilis* ATCC29413 (NC_007413).

**Set 4** (Average G+C  = 49.4%): *Marinobacter aquaeolei* VT8 (NC_008740), *Salinibacter ruber* DSM13855 (NC_007677), *Protochlamydia amoebophila* UWE25 (NC_005861), *Treponema denticola* ATCC35405 (NC_002967), *Thermosynechococcus elongatus* BP-1 (NC_004113).

### Exhaustive samping of polypurine oligomers

The located oligonucleotides were then aligned using the ClustalW multiple sequence alignment algorithm [19] in Jalview [20], trimmed, and condensed into respective sequence logos with Weblogo [21].

All sets contained polypurine/polypyrimidine tracts with some variation. Pure A-tracts were rare so introduced guanine (or for pyrimidine tracts cytosine) nucleotides were taken into account. Thus all 512 possible polypurine and polypyrimidine octamers were extracted from the dataset. These candidate abundant oligomers were then subjected to a further control for overrepresentation using a second order Markov model (below).

### Controlling for embedded oligomers

Multiple occurrences of a shorter constituent oligonucleotide might lead to an oligomer being apparently overrepresented, respective to a random counterpart, due to the zero-order Markov model methodology. That is, overrepresentation of a given longer oligomer may result simply from conservation of shorter embedded oligomers. Overrepresentation was thus confirmed via a second-order Markov model. These models are based on trinucleotides, so factor out the effects of embedded mono- and dinucleotides.

All 684 microbial chromosomes were then scanned for the 512 possible polypurine and polypyrimidine octamers using a second order Markov model in the program R'MES [22] with a Gaussian distribution for frequent oligomers and otherwise default parameters. Results were filtered into over- and underrepresented sets for each oligomer. Thereafter an overrepresentation index was created subtracting genomes which the oligo was underrepresented in. When compared by rank number, results were in close agreement with those generated from zero-order Markov models for the same dataset. Not only the number of genomes oligomers are overrepresented in, but the copy numbers (as opposed to overrepresentation alone) are relevant to our goal of finding widespread and abundant oligomers. Thus oligomers were sorted by zero-order Markov model rank, as this dataset is further restricted by oligomers per megabase whereas the second-order Markov model dataset is not.

### Analysis of oligomers in coding regions

Percentages of genomes from the February 2008 NCBI RefSeq genome collection which are coding were calculated with an in-house script (available from the authors). The percentage of abundant oligomers also occurring in coding regions was then calculated. A further script calculated the coding frame which each oligomer within an ORF was present in using genome position and annotation information. Figures were plotted using the statistical environment R [23].

## Results

Sequenced bacterial and archaeal genomes were scanned for globally overrepresented 8- to 14-mers by zero-order and second-order Markov models. The search revealed a highly related set of homopurine and homopyrimidine octanucleotides as the statistically most overrepresented widespread oligomers. Normalisation was performed for base composition and embedded oligomers in two distinct analyses. Table 1 lists the 15 most common octamers. Nonamers and longer oligomers were not found to be in excess of the implemented thresholds. According to the threshold criteria the six most common homopurines contain one, two or three guanine nucleosides, while the nine most common homopyridimines carry one to four cytosine nucleosides. These 15 octanucleotides occur six- to twentyfold more frequently in a dataset of 684 chromosomes than expected for a randomly selected octanucleotide (Figure 1).

**Table 1:** The most widespread overrepresented oligomers according to two different methods and data on their overrepresentation.

	Number of chromosomes oligomer overrepresented in (n = 684)		
Oligo	OligoCounter zero order Markov model (rank)	Overrepresented and not underrepresented by 2nd Order Markov model (rank)	Median copy number per megabase (quartiles)
GAAGAAGA	489 (1)	665 (1)	59 (43 – 80)
TCTTCTTC	483 (2)	665 (2)	60 (43 – 80)
AAGAAAAA	404 (3)	531 (13)	171 (79 – 230)
TTTTTCTT	400 (4)	528 (14)	165 (77 – 228)
AAAGAAAA	382 (5)	470 (29)	166 (84 – 238)
TTTTCTTC	376 (6)	514 (16)	92 (57 – 134)
GAAGAAAA	374 (7)	503 (21)	94 (55 – 133)
TTTTCTTT	371 (8)	462 (34)	166 (86 – 235)
AGAAAAAG	367 (9)	507 (17)	109 (71 – 135)
GAAAAAGA	365 (10)	516 (15)	110 (69 – 141)
TCTTTTTC	365 (11)	504 (19)	110 (69 – 144)
CTTTTTCT	361 (12)	500 (23)	106 (69 – 137)
CTTCTTCT	361 (13)	655 (3)	66 (48 – 89)
CCTTCTTC	357 (14)	605 (8)	47 (40 – 56)
TTTCTTTT	356 (15)	379 (60)	166 (93 – 244)

Small differences in the number of genomes oligomers are overrepresented in are due to strand biases.

Individual prokaryotic genomes harbour between a few dozen to up to 700 copies of each of the 15 octanucleotides in one megabase of sequence (Figure 1). The octamer 5′-GAAGAAGA and its reverse complement 5′-TCTTCTTC were the two most widespread octanucleotides that, according to second - order Markov chain analysis, were overrepresented in 97% of analysed bacterial and archaeal chromosomes (Figure 1, Table 1).

**Figure 2:**
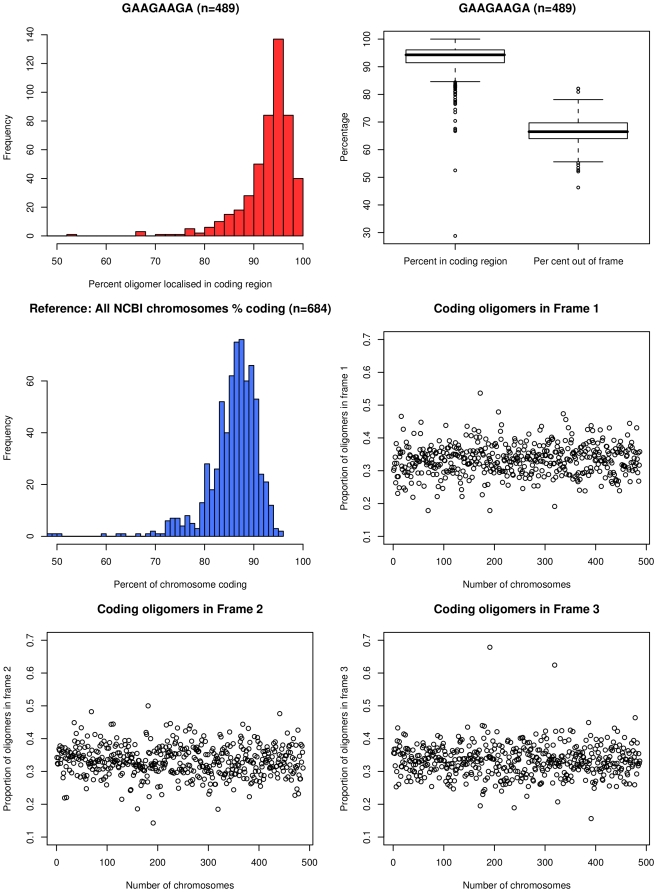
Localisation in coding and non-coding regions. Localisation of abundant oligomers in coding regions and individual coding frames. The oligomer and the number of chromosomes it is found in are listed in the title of the top left graph. This histogram shows the distribution, in red, of chromosomes where this oligomer is present in coding regions (as a percentage of all occurrences of the oligomer). This histogram can be compared and contrasted with the distribution of percentage of genomic coding regions across all 684 chromosomes used in the analysis, which is presented in a blue histogram below. On the top right a box and whisker plot displays the localisation in coding regions of this oligomer across all chromosomes in which it is found, and the percentage of occurrences which are not in the translated reading frame. The remaining three scatter plots (middle right, bottom left and right) show the proportion of the oligomers in reading frames 1, 2, and 3 respectively. Frame 1 is considered “in frame”. Together, these figures demonstrate the lack of bias of these oligomers towards any particular reading frame in the chromosomes in which they are overrepresented.

**Figure 3:**
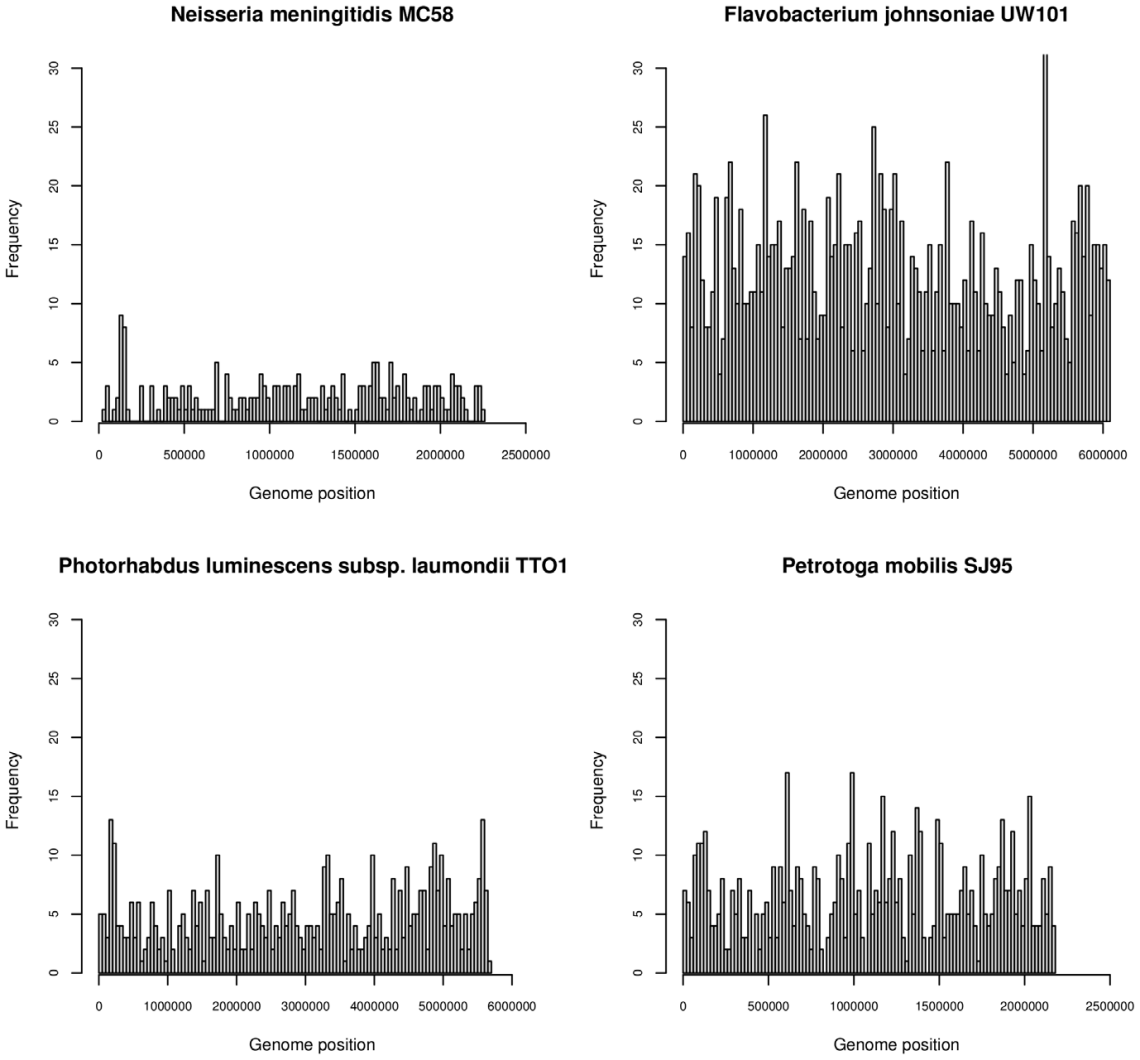
Oligomers do not cluster at particular genomic positions. Distribution of the oligomer AAGAAAAA in four genomes from the Betaproteobacteria, Gammaproteobacteria, Bacteroidetes, and Thermotogae. No distinct clusters of this oligomer are present, rather they are distributed throughout the genome. Similar distributions were also observed in other genomes.

In more than 90% of bacterial genomes the coding sequence makes up 80% or more of total sequence (mean 86%) (Figure 2). The frequency of the 15 abundant octamers in non-coding and coding sequences roughly matched the distribution of coding and non-coding chromosomal sequence in bacterial genomes (Figure 2, Supplementary Information, Figure S1). The distribution, however, was broader and showed a bias towards coding and non-coding sequence for six and five octamers, respectively (Figures 2, S1). A preponderance of individual oligomers in either non-coding or coding sequences was seen in individual chromosomes, but no global trend for the localization of any of the most widespread octamers in coding or non-coding sequence was noted. All 15 octamers were moreover randomly distributed between the three reading frames of coding sequence (Figure S1) implying that neither codon usage bias nor highly common tripeptides [3] account for the high frequency of the oligomers. The 15 abundant oligomers were evenly distributed along the individual genomes. No clusters were observed in the chromosomes at the resolution of 50 kB as illustrated by the four examples shown in Figure 3.

The 15 octanucleotides were found to be overrepresented in chromosomes of nearly all phylogenetic groups, but a few taxonomic exceptions were noted (Table S1). All yet sequenced acidobacteria (two chromosomes), deinococci-thermi (five) and planctomycetes (one) carried most oligomers at frequencies below 31 per Mb (Table S1), indicating that these homopurines and homopyrimidines have not been positively selected in these clades. All but the two most abundant octamers 5′-GAAGAAGA and 5′-TCTTCTTC were not overrepresented in numerous actinobacteria, alpha- and beta-proteobacteria (Table S2).

## Discussion

Oligonucleotide usage is not neutral because of structural, functional, biological and coding constraints [2], [4], [5], [9]. Since the abundant octanucleotides show no preference for any position in the reading frame (Figures 2, S1), their abundance most likely does not reflect any transcriptional or translational demands, but rather results from structural and functional features of the oligomer within the chromosome.

Structural properties of oligonucleotides have been predicted from computational models [24], [25] that are based on the crystal structures of double helical DNA oligomers [26], experimental studies on DNA flexibility and bending [27], empirical energy functions [28], and quantum mechanical calculations [29]. Experiments in solution are informative with regard to the overall conformation such as the superhelical radius and the helical repeat ( =  number of base-pairs per turn), but precise information about the local structure of DNA at the dinucleotide step level has only been obtained from NMR studies and X-ray crystallography [30]–[32]. Relevant structural scales are slide and propeller twist. Slide as a measure of flexibility is the relative displacement of one base-pair to its neighbour along the direction of the long axis in a dinucleotide step. Propeller twist is a twist about the long axis that makes the two bases of a pair non-coplanar.

The structural scales derived from solved crystal structures of naked DNA oligomers assign high propeller twist (−20°) and low flexibility (0.3 Å) to the AA/TT dinucleotide steps in the 15 abundant homopurines and homopyrimidines found in our analysis, whereas the GA/CT and AG/CT steps should show intermediate conformational flexibility (−13°, 0.7 Å) [31], [33]. Importantly, the three dinucleotide steps present in the 15 widespread oligomers have a more or less unique value of slide whereas the other seven dinucleotide steps that all are absent from our set of 15 octanucleotides show a wide range of slide. In other words, the 15 abundant octanucleotides share the structural feature that the conformation of the individual base-pairs in each oligomer duplex should be largely independent of the sequence context, because the conformational properties of all possible neighbouring steps are known to be compatible [25]. Thus, each copy of the octanucleotide in the chromosome should exhibit a highly similar three-dimensional structure supporting our interpretation that the widespread octanucleotides represent structural signals.

Five of the six widespread homopurines and six of the nine homopurines that are complementary to the widespread homopyrimidines harbour a A_4_ or A_5_ tract. These A-tract regions are known to show cooperative transition to a structure more resembling the structure of poly(dA) · poly(dT) [34]–[36]. The structure of poly(dA) · poly(dT) is distinct from that of canonical B-DNA [37], [38]. The minor groove is narrow and the bases are highly propeller twisted and negatively inclined relative to the overall helix axis. When A-tracts are repeated in tandem with the helical repeat, the sequence elements are placed along the same side of the double helix, so that they accumulate coherently to yield macroscopic curvature [32], [39]. A-tracts are the main sequence elements that lead to intrinsic DNA bending [40], and have been found to be abundant in prokaryotic coding regions [39]. While DNA curvature due to A-tracts is likely to be locally important in the formation of local loops, it is unable to account for the degree of compaction seen in the supercoiled prokaryotic nucleoid [39]. A-tracts are however known to be associated with DNA-binding proteins [41], [42]. Many of these proteins target the abnormally narrow minor groove associated with A-tracts and are known to play a role in nucleoid packaging [16], [43]. These proteins include the essential histone like protein HU and nucleoid structuring protein H-NS [41], [44]. The H-NS binding sites includes consensus A-tracts [44], and HU recognises its target by ‘indirect readout’ of structural parameters [41]. Arginine residues have been recently demonstrated to play a key role in these protein-DNA contacts [16]. Furthermore, considerable evidence links eukaryotic A-tracts to nucleosome positioning [16], [42]. As such, some limited putative commonalities exist on the DNA sequence and structural level between packaging of prokaryotic and eukaryotic chromosomes [43].

A-tracts fulfill a variety of functions in vivo not all connected with intrinsic DNA bending per se but rather with the unusual structural properties of A-tracts [32]. For example, A-tracts are localized in terminal loops of superhelical domains [32], play a role in transcriptional regulation [44], DNA replication and recombination and are involved in eukaryotes in the global positioning of nucleosomes via nucleosome exclusion [45], [46]. The fact that A-tracts are ‘multitasking’ DNA elements may explain why they are common in the 15 most widespread octanucleotides in prokaryotes.

A further feature of homopurines and homopyrimidines is their ability to form a triple-stranded helix [47]–[51]. One homopyrimidine tract forms conventional Watson-Crick base-pairs with the homopurine tract and the second homopyrimidine strand is Hoogsteen base-paired in the major groove to the homopurine strand. Two complementary homopurine-homopyrimidine octanucleotides are sufficient to induce this phenomenon [49]. Triple helix formation is known to be disfavoured in pure oligo-dA tracts, but the insertion of a single central guanine nucleoside has been shown to lead to observable triplex formation at neutral pH [51]. It is interesting to note that neither oligo(dA)_8_ nor oligo(dT)_8_ belong to the most common octanucleotides. The 15 widespread oligomers carry one or more guanines or cytidines, respectively, implying that the potential triple helices may putatively be stable in bacteria at physiological temperature (20°C) and intracellular osmolarity and magnesium concentrations. Triple helix formation in octanucleotides has been demonstrated by NMR [49]. Thus short stretches of triplex DNA or hybrids of RNA with duplex DNA could possibly exist in numerous archaeal and bacterial chromosomes, at least in mesophilic and psychrophilic microorganisms growing at lower temperatures where triplexes are more stable.

Our finding that the most widespread octanucleotides are homopurines and homopyrimidines was not unexpected. More than ten years ago Deschavanne and co-workers [7] reported that in a dataset of five bacterial genomes the abundant penta- to octanucleotides were composed of purine and pyrimidine stretches. At that time the number of completely sequenced bacterial genomes was scarce. Genome sequences are now available from all major clades and hence we could demonstrate the overrepresentation of a set of homopurines and homopyrimidines as a global phenomenon in bacteria. Exceptions do exist, particularly for bacteria with high G+C contents. These bacteria may use different mechanisms to those mediated by A-tracts for DNA packaging [39].

For bacterial organisms that are not closely related to each other, the presence/absence of oligonucleotides of intermediate length are not correlated [52]. In this respect the most widespread statistically overrepresented octanucleotides in archaea and bacteria are the exception to the rule. These homopurine: homopyrimidine strings are characterised by low conformational flexibility, exhibit a structure that is distinct from that of canonical B-DNA and may possess the ability to form triple helices. Their most likely functional role appears to be related to local bending and possible binding sites for DNA packaging proteins such as HU [43]. These proteins recognise the narrow minor groove which is associated with A-tracts [16]. Conservation of these oligomers in diverse taxonomic lineages implies an early evolutionary origin.

## Supporting Information

Figure S1 Localisation in coding and non-coding regions. Localisation of abundant oligomers in coding regions and individual coding frames. The oligomer and the number of chromosomes it is found in are listed in the title of the top left graph. This histogram shows the distribution, in red, of chromosomes where this oligomer is present in coding regions (as a percentage of all occurrences of the oligomer). This histogram can be compared and contrasted with the distribution of percentage of genomic coding regions across all 684 chromosomes used in the analysis, which is presented in a blue histogram below. On the top right a box and whisker plot displays the localisation in coding regions of this oligomer across all chromosomes in which it is found, and the percentage of occurrences which are not in the translated reading frame. The remaining three scatter plots (middle right, bottom left and right) show the proportion of the oligomers in reading frames 1, 2, and 3 respectively. Frame 1 is considered “in frame”. Together, these figures demonstrate the lack of bias of these oligomers towards any particular reading frame in the chromosomes in which they are overrepresented.
(1.35 MB PDF)

Table S1 Prokaryotic lineages lacking the top 15 overrepresented abundant oligomers. N indicates the number of genomes in the lineage.
(0.29 MB PDF)

Table S2 Raw data showing the number of genomes each of the homopurines and homopyrimidines are overrepresented and underrepresented in.
(0.15 MB XLS)
