# Supplementary material for: Abundant Oligonucleotides Common to Most Bacteria
Source: PLoS One. 2010 Mar 23;5(3):e9841. doi: 10.1371/journal.pone.0009841 (PMC2843746; doi:10.1371/journal.pone.0009841)
Supplement: Figure S1 — Localisation in coding and non-coding regions. Localisation of abundant oligomers in coding regions and individual coding frames. The oligomer and the number of chromosomes it is found in are listed in the title of the top left graph. This histogram shows the distribution, in red, of chromosomes where this oligomer is present in coding regions (as a percentage of all occurrences of the oligomer). This histogram can be compared and contrasted with the distribution of percentage of genomic coding regions across all 684 chromosomes used in the analysis, which is presented in a blue histogram below. On the top right a box and whisker plot displays the localisation in coding regions of this oligomer across all chromosomes in which it is found, and the percentage of occurrences which are not in the translated reading frame. The remaining three scatter plots (middle right, bottom left and right) show the proportion of the oligomers in reading frames 1, 2, and 3 respectively. Frame 1 is considered “in frame”. Together, these figures demonstrate the lack of bias of these oligomers towards any particular reading frame in the chromosomes in which they are overrepresented. (1.35 MB PDF) [file pone.0009841.s001.pdf]

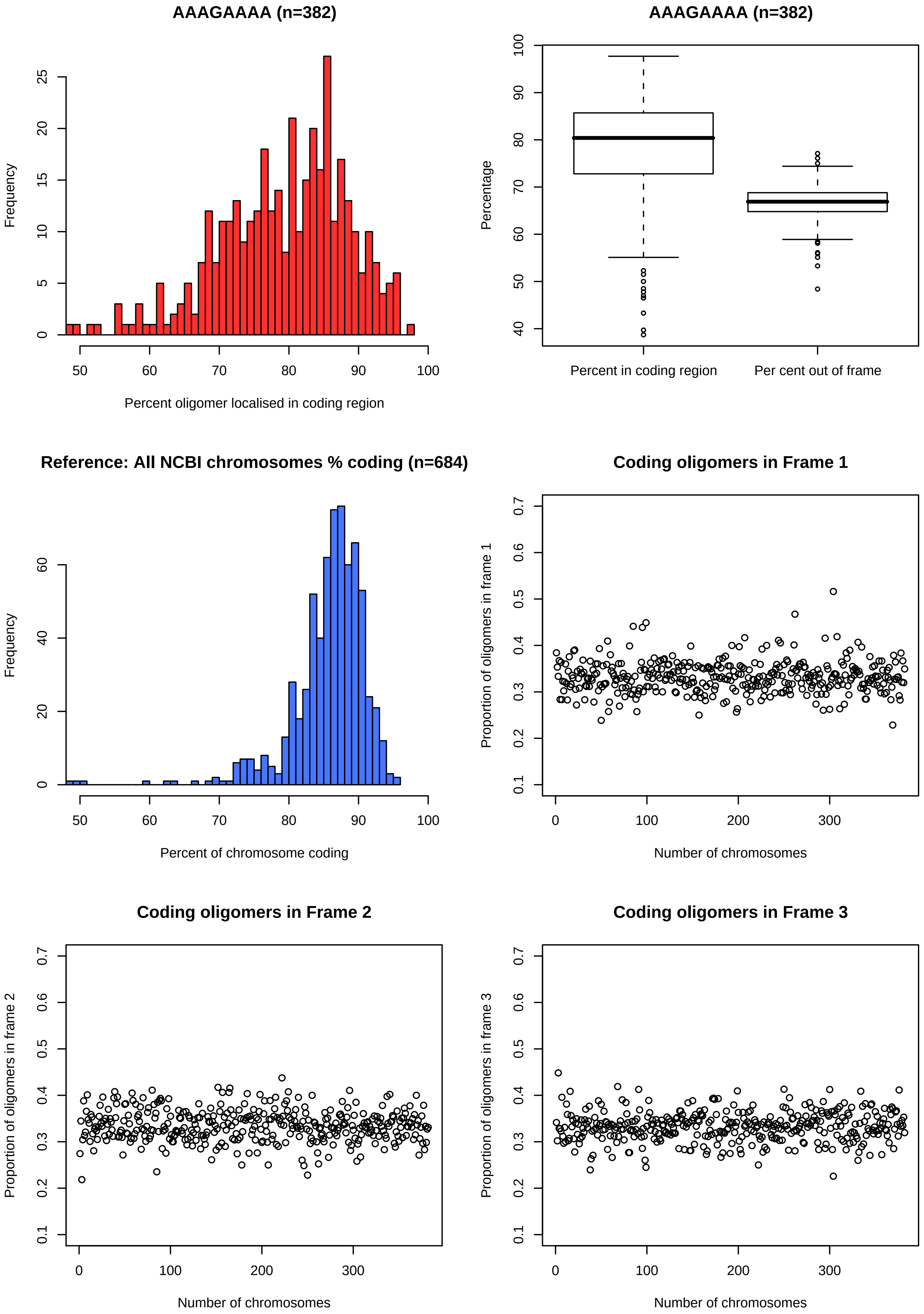
AAAGAAAA (n=382)
Frequency
Percent oligomer localised in coding region
AAAGAAAA (n=382)
Percentage
Percent in coding region
Per cent out of frame
Reference: All NCBI chromosomes % coding (n=684)
Frequency
Percent of chromosome coding
Coding oligomers in Frame 1
Proportion of oligomers in frame 1
Number of chromosomes
Coding oligomers in Frame 2
Proportion of oligomers in frame 2
Number of chromosomes
Coding oligomers in Frame 3
Proportion of oligomers in frame 3
Number of chromosomes

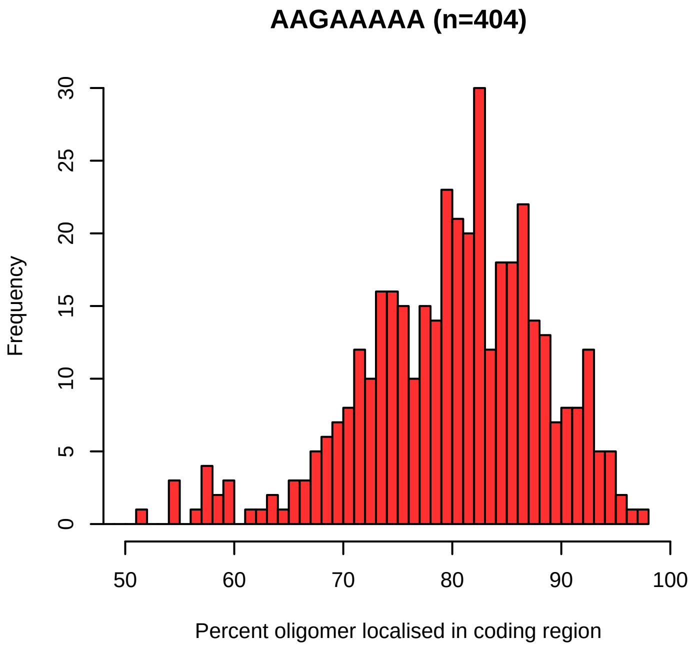
AAGAAAAA (n=404)
Frequency
Percent oligomer localised in coding region

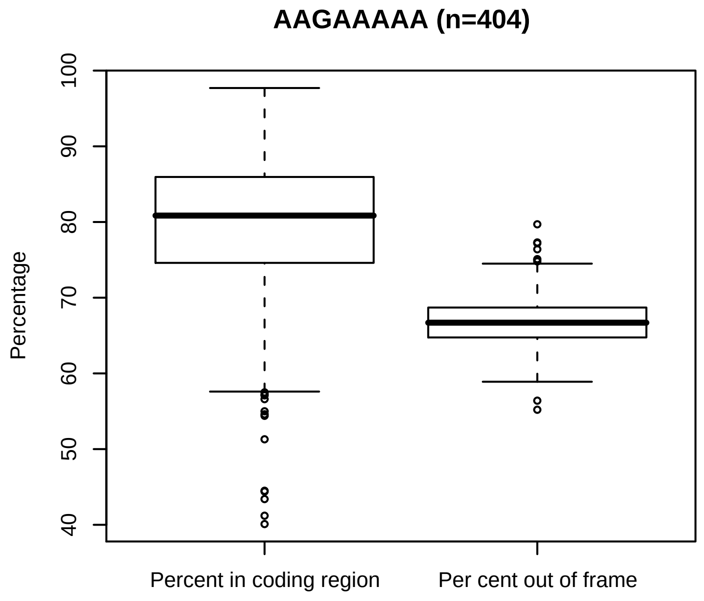
AAGAAAAA (n=404)
Percentage
Percent in coding region
Per cent out of frame

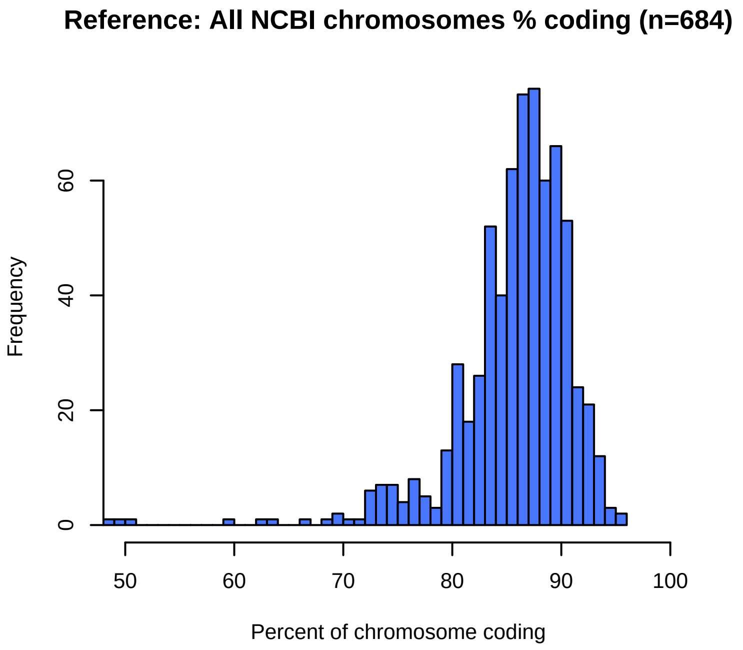
Reference: All NCBI chromosomes % coding (n=684)
Frequency
Percent of chromosome coding

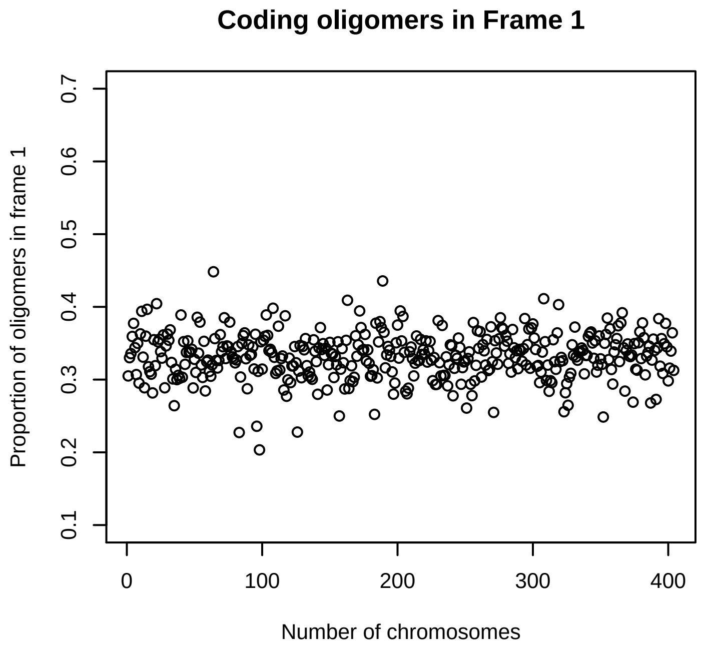
Coding oligomers in Frame 1
Proportion of oligomers in frame 1
Number of chromosomes

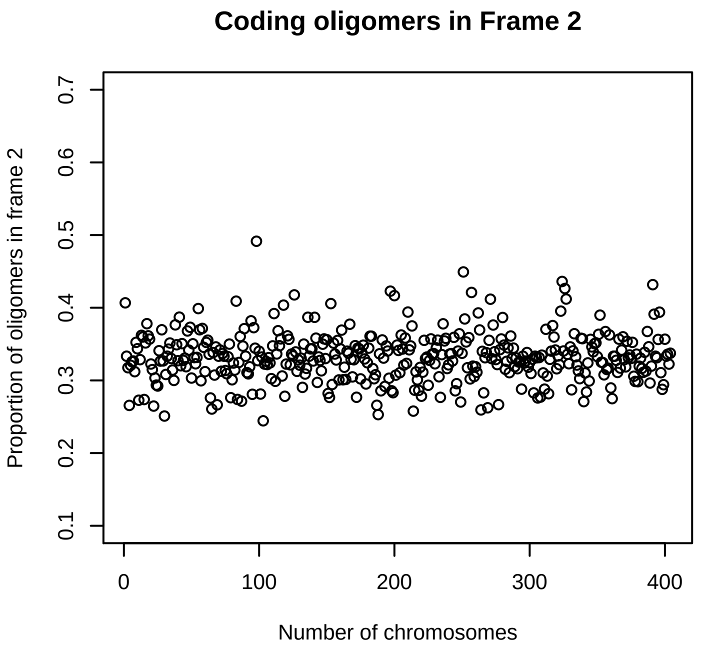
Coding oligomers in Frame 2
Proportion of oligomers in frame 2
Number of chromosomes

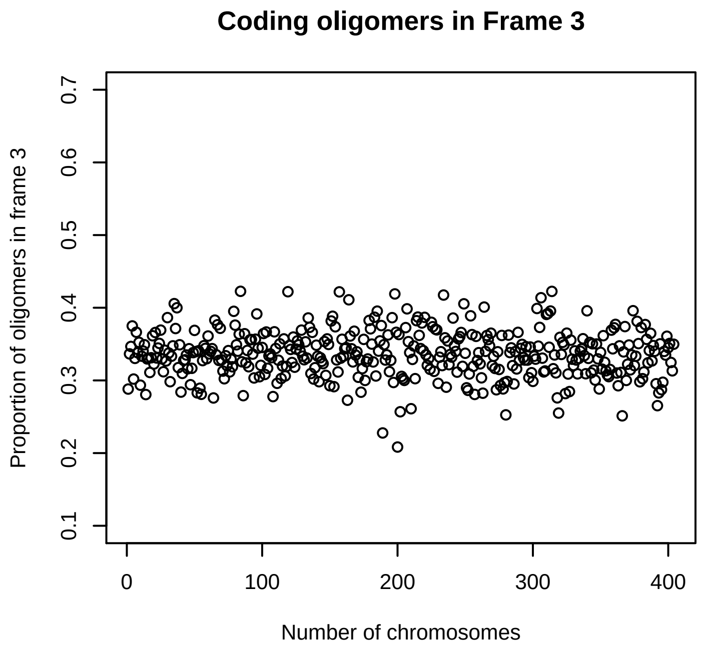
Coding oligomers in Frame 3
Proportion of oligomers in frame 3
Number of chromosomes

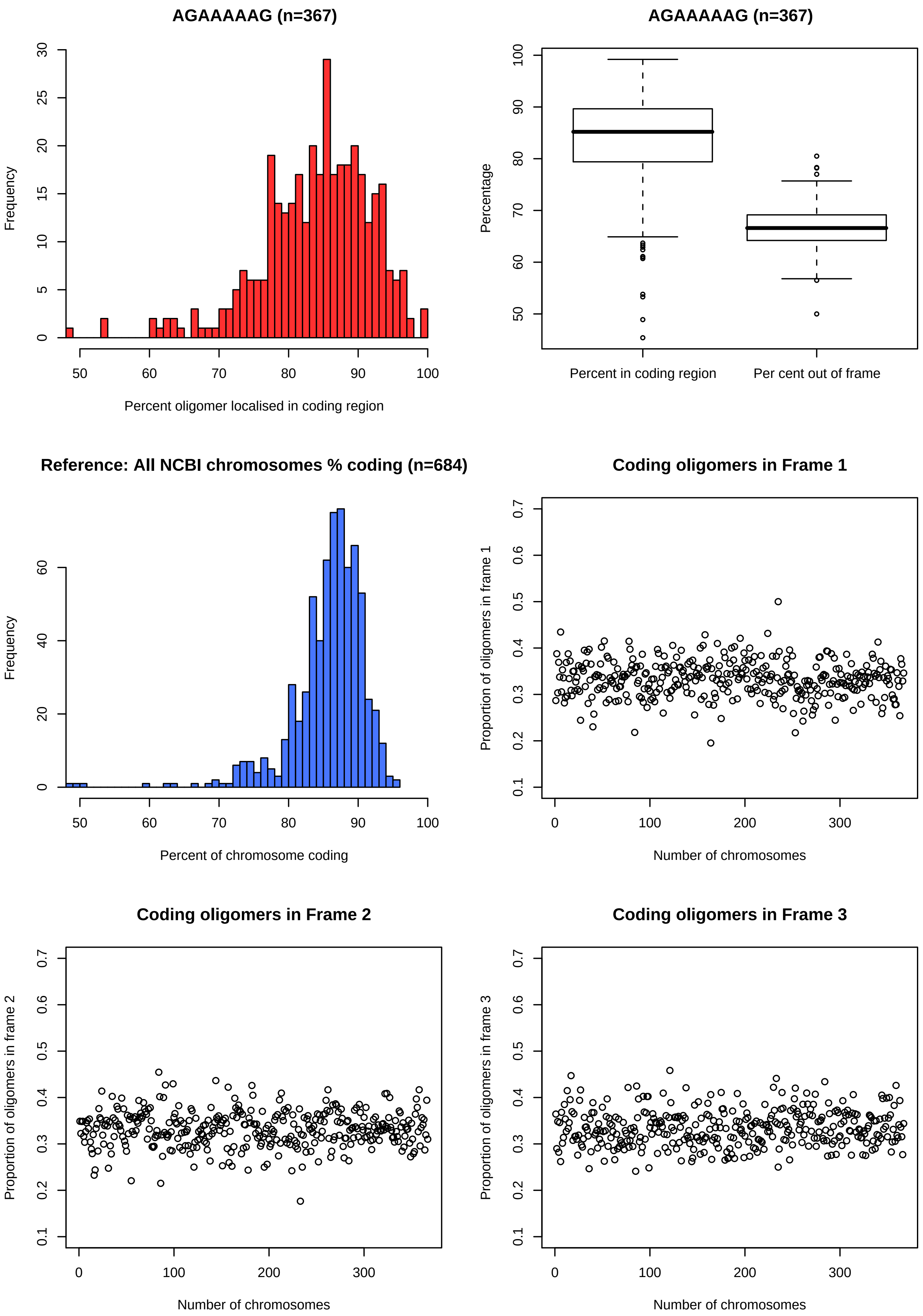
AGAAAAAG (n=367)
Frequency
Percent oligomer localised in coding region
0 5 10 15 20 25 30
50 60 70 80 90 100
AGAAAAAG (n=367)
Percentage
50 60 70 80 90 100
Percent in coding region
Per cent out of frame
Reference: All NCBI chromosomes % coding (n=684)
Frequency
0 20 40 60
50 60 70 80 90 100
Percent of chromosome coding
Coding oligomers in Frame 1
Proportion of oligomers in frame 1
0.1 0.2 0.3 0.4 0.5 0.6 0.7
0 100 200 300
Number of chromosomes
Coding oligomers in Frame 2
Proportion of oligomers in frame 2
0.1 0.2 0.3 0.4 0.5 0.6 0.7
0 100 200 300
Number of chromosomes
Coding oligomers in Frame 3
Proportion of oligomers in frame 3
0.1 0.2 0.3 0.4 0.5 0.6 0.7
0 100 200 300
Number of chromosomes

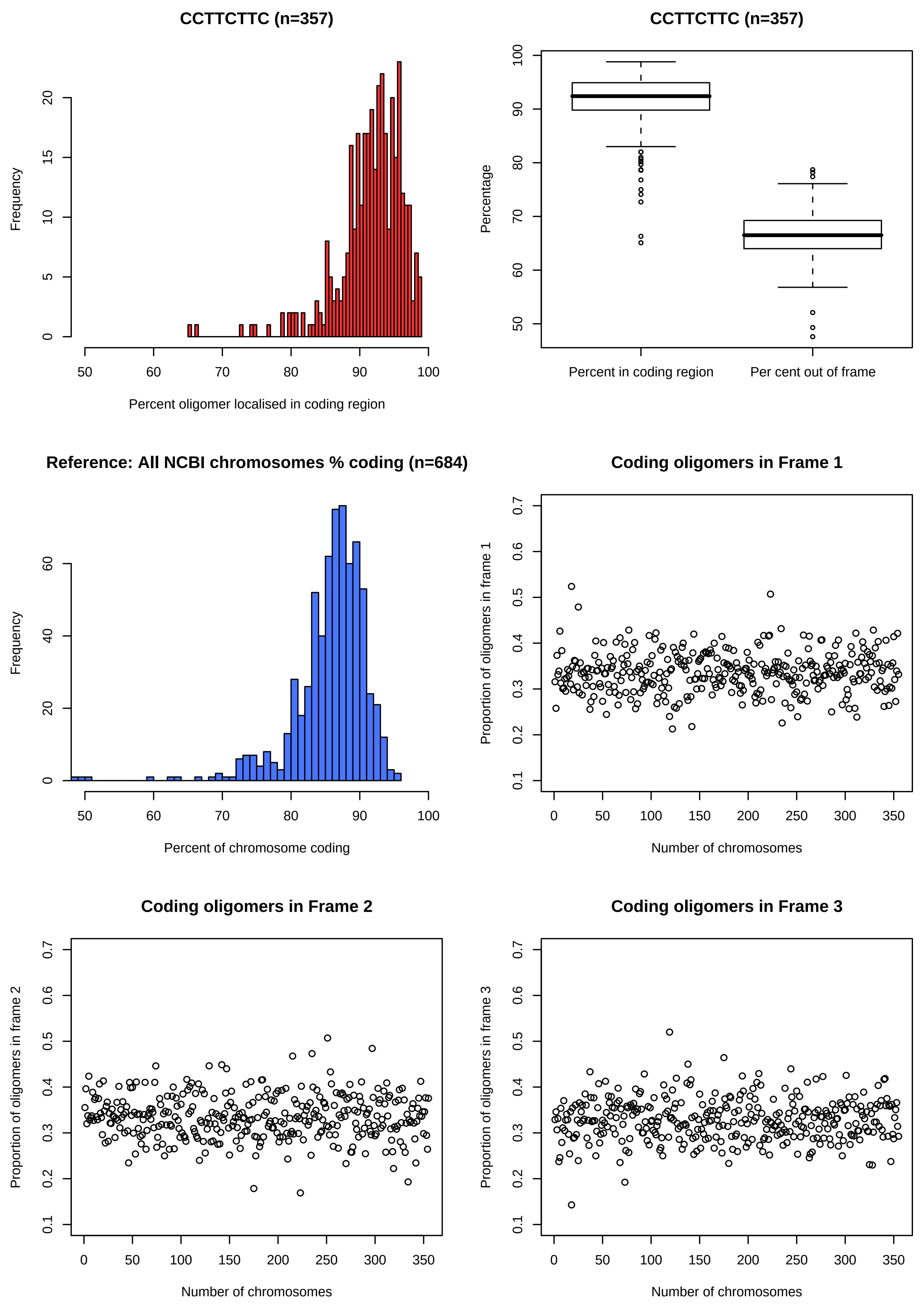
CCTTCTTC (n=357)
Frequency
Percent oligomer localised in coding region
0
5
10
15
20
50
60
70
80
90
100
CCTTCTTC (n=357)
Percentage
50
60
70
80
90
100
Percent in coding region
Per cent out of frame
Reference: All NCBI chromosomes % coding (n=684)
Frequency
Percent of chromosome coding
0
20
40
60
50
60
70
80
90
100
Coding oligomers in Frame 1
Proportion of oligomers in frame 1
Number of chromosomes
0.1
0.2
0.3
0.4
0.5
0.6
0.7
0
50
100
150
200
250
300
350
Coding oligomers in Frame 2
Proportion of oligomers in frame 2
Number of chromosomes
0.1
0.2
0.3
0.4
0.5
0.6
0.7
0
50
100
150
200
250
300
350
Coding oligomers in Frame 3
Proportion of oligomers in frame 3
Number of chromosomes
0.1
0.2
0.3
0.4
0.5
0.6
0.7
0
50
100
150
200
250
300
350

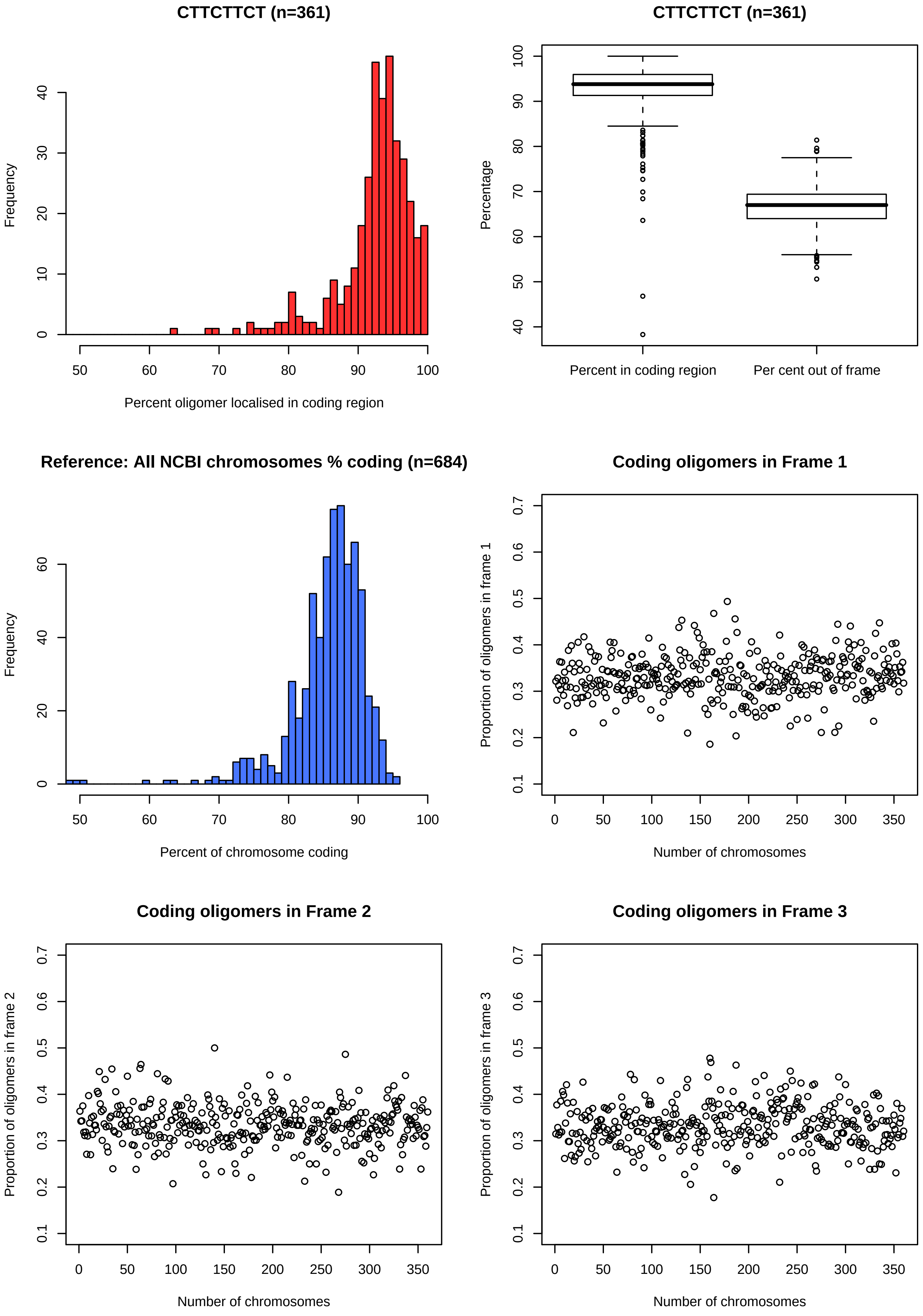
CTTCTTCT (n=361)
Frequency
Percent oligomer localised in coding region
CTTCTTCT (n=361)
Percentage
Percent in coding region
Per cent out of frame
Reference: All NCBI chromosomes % coding (n=684)
Frequency
Percent of chromosome coding
Coding oligomers in Frame 1
Proportion of oligomers in frame 1
Number of chromosomes
Coding oligomers in Frame 2
Proportion of oligomers in frame 2
Number of chromosomes
Coding oligomers in Frame 3
Proportion of oligomers in frame 3
Number of chromosomes

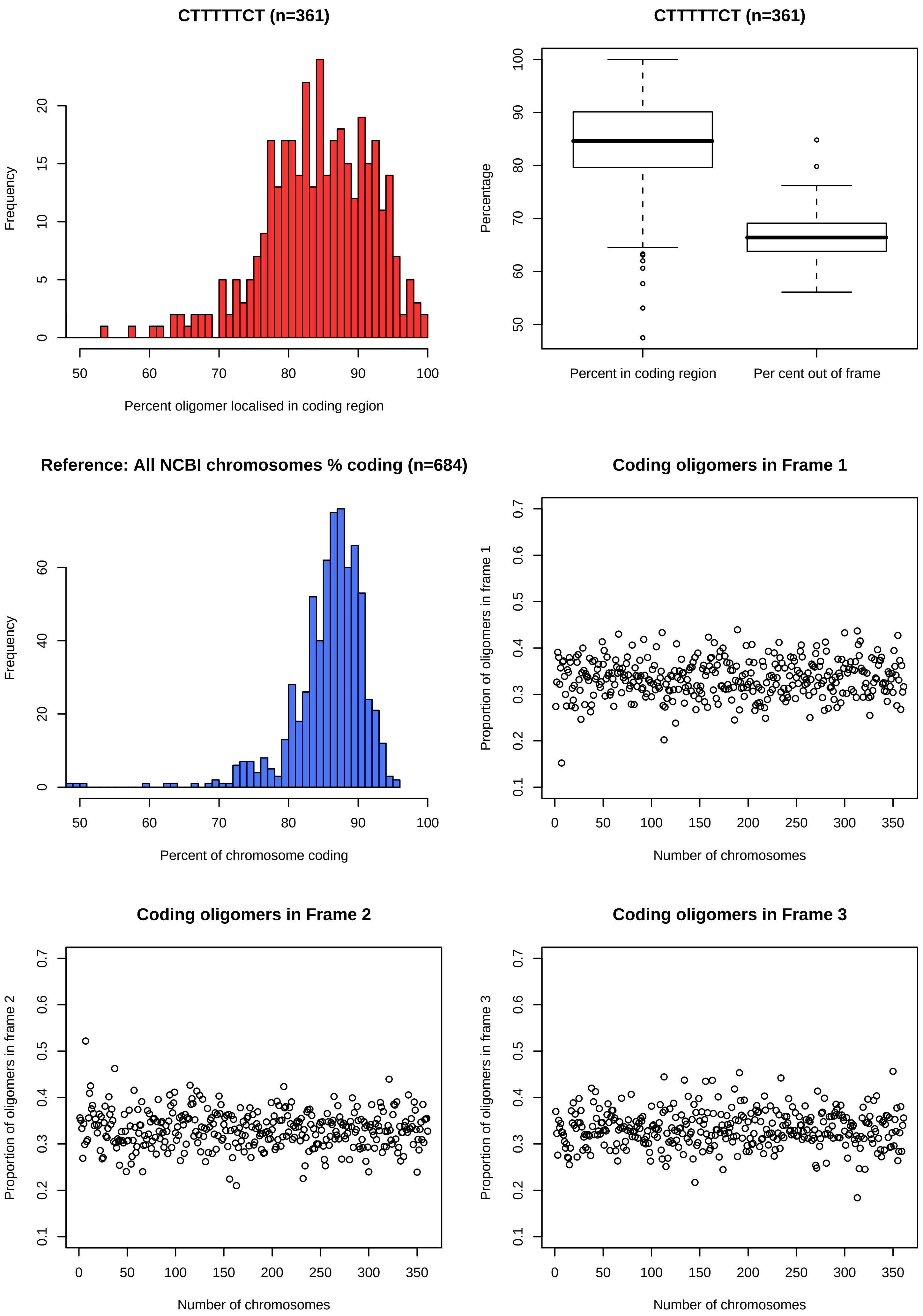
CTTTTTCT (n=361)
Frequency
Percent oligomer localised in coding region
CTTTTTCT (n=361)
Percentage
Percent in coding region
Per cent out of frame
Reference: All NCBI chromosomes % coding (n=684)
Frequency
Percent of chromosome coding
Coding oligomers in Frame 1
Proportion of oligomers in frame 1
Number of chromosomes
Coding oligomers in Frame 2
Proportion of oligomers in frame 2
Number of chromosomes
Coding oligomers in Frame 3
Proportion of oligomers in frame 3
Number of chromosomes

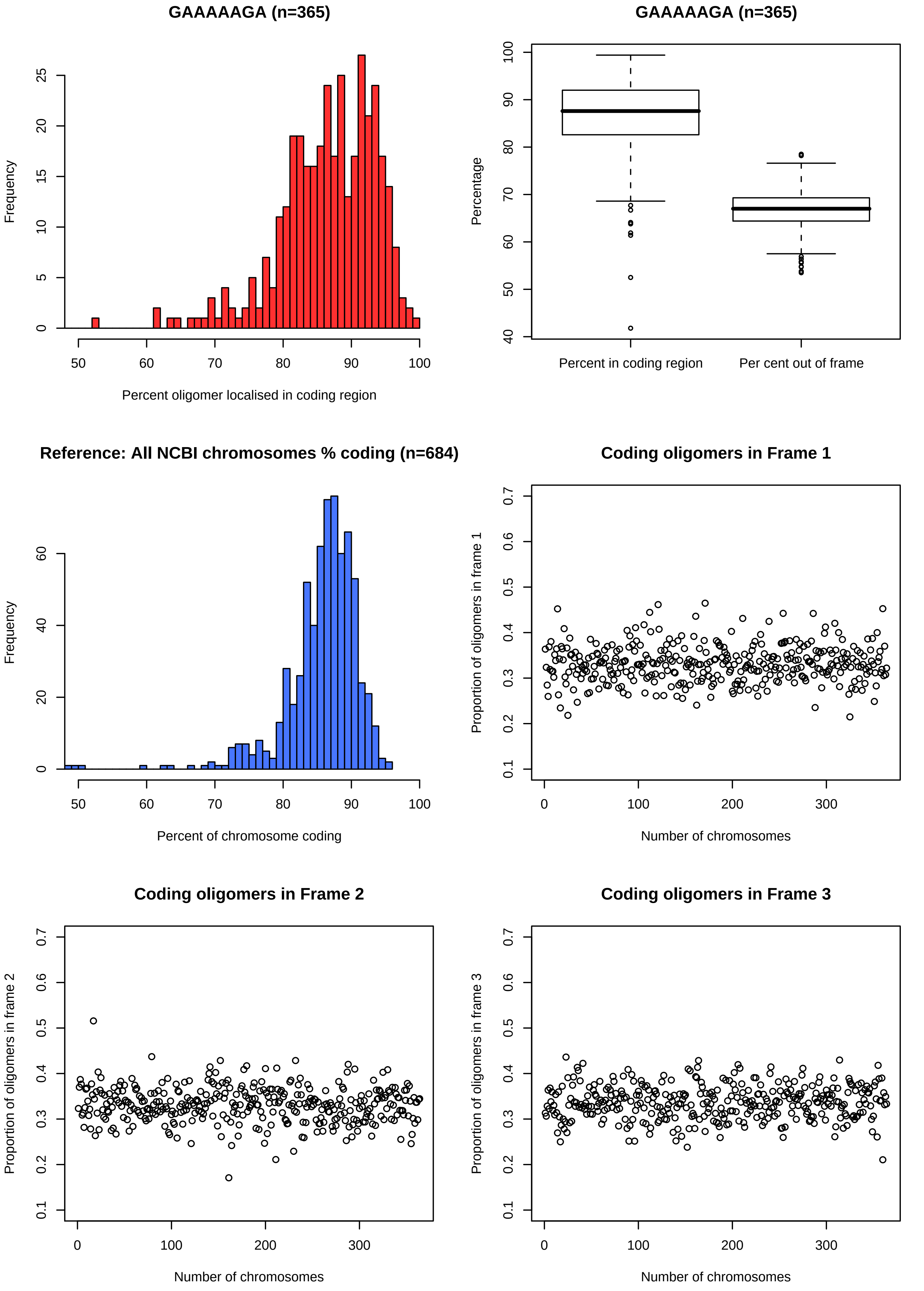
GAAAAAGA (n=365)
Frequency
Percent oligomer localised in coding region
GAAAAAGA (n=365)
Percentage
Percent in coding region
Per cent out of frame
Reference: All NCBI chromosomes % coding (n=684)
Frequency
Percent of chromosome coding
Coding oligomers in Frame 1
Proportion of oligomers in frame 1
Number of chromosomes
Coding oligomers in Frame 2
Proportion of oligomers in frame 2
Number of chromosomes
Coding oligomers in Frame 3
Proportion of oligomers in frame 3
Number of chromosomes

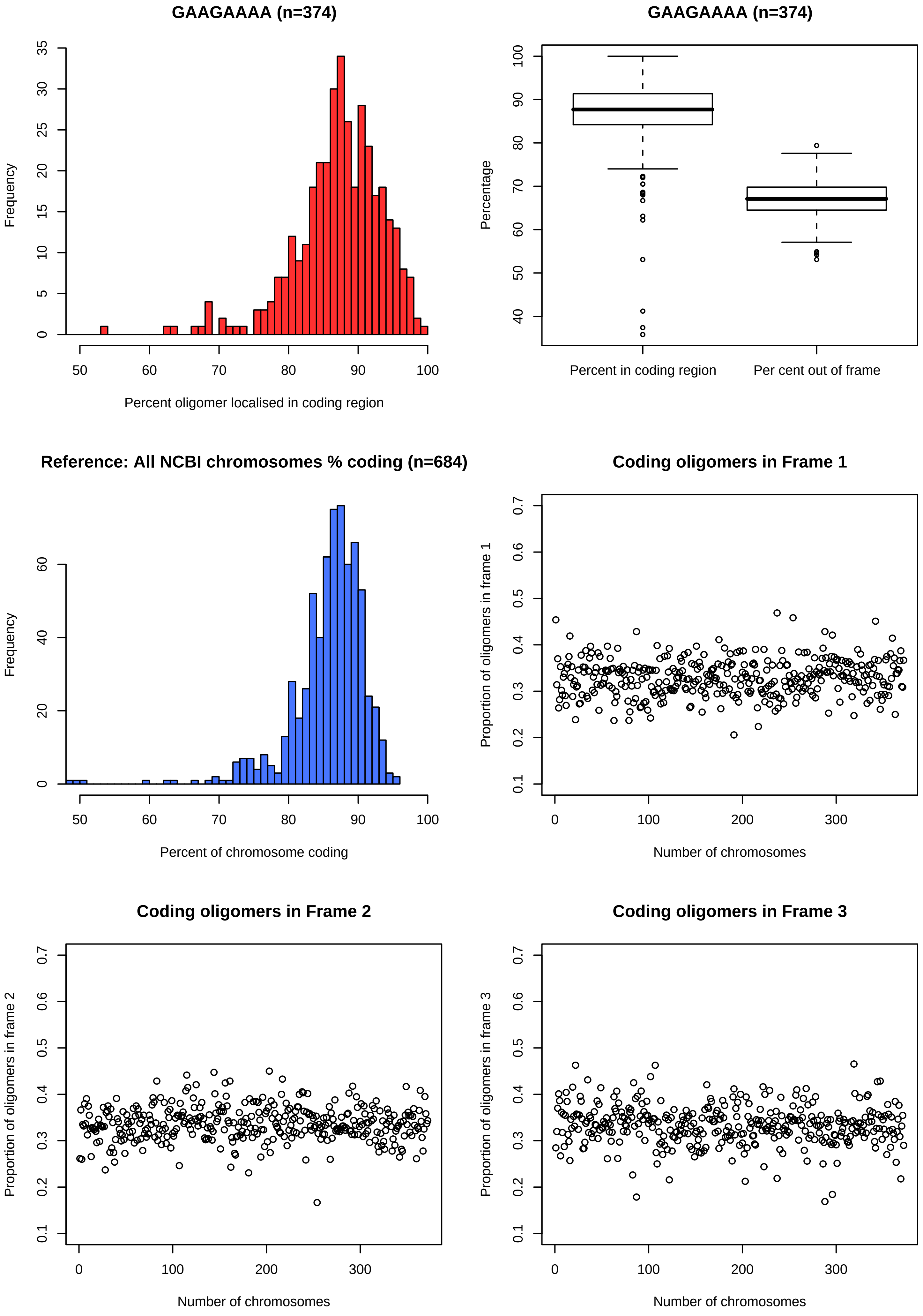
GAAGAAAA (n=374)
Frequency
Percent oligomer localised in coding region
GAAGAAAA (n=374)
Percentage
Percent in coding region
Per cent out of frame
Reference: All NCBI chromosomes % coding (n=684)
Frequency
Percent of chromosome coding
Coding oligomers in Frame 1
Proportion of oligomers in frame 1
Number of chromosomes
Coding oligomers in Frame 2
Proportion of oligomers in frame 2
Number of chromosomes
Coding oligomers in Frame 3
Proportion of oligomers in frame 3
Number of chromosomes

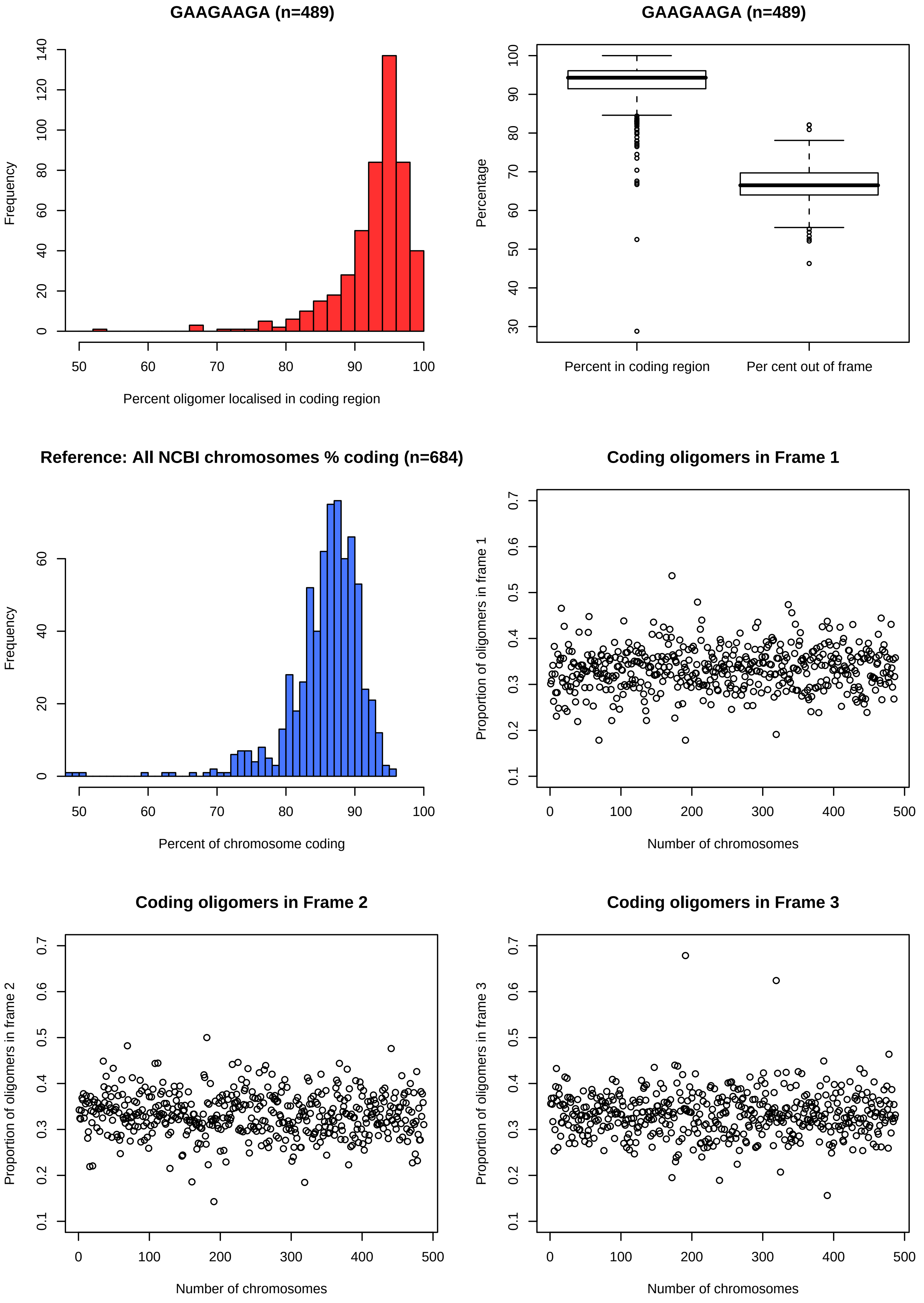
GAAGAAGA (n=489)
Frequency
Percent oligomer localised in coding region
GAAGAAGA (n=489)
Percentage
Percent in coding region
Per cent out of frame
Reference: All NCBI chromosomes % coding (n=684)
Frequency
Percent of chromosome coding
Coding oligomers in Frame 1
Proportion of oligomers in frame 1
Number of chromosomes
Coding oligomers in Frame 2
Proportion of oligomers in frame 2
Number of chromosomes
Coding oligomers in Frame 3
Proportion of oligomers in frame 3
Number of chromosomes

TCTTCTTC (n=483)

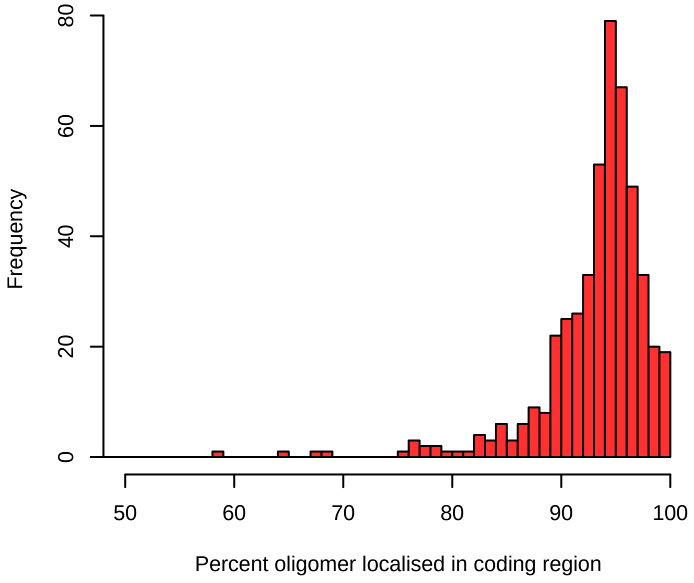

TCTTCTTC (n=483)

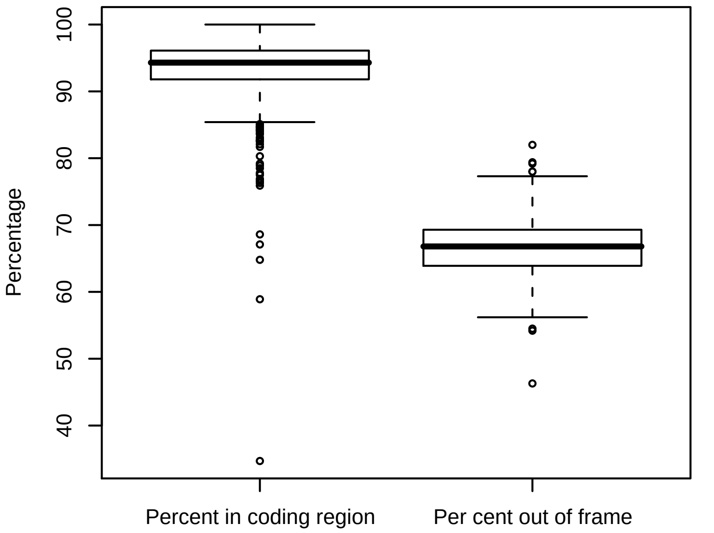

Reference: All NCBI chromosomes % coding (n=684)

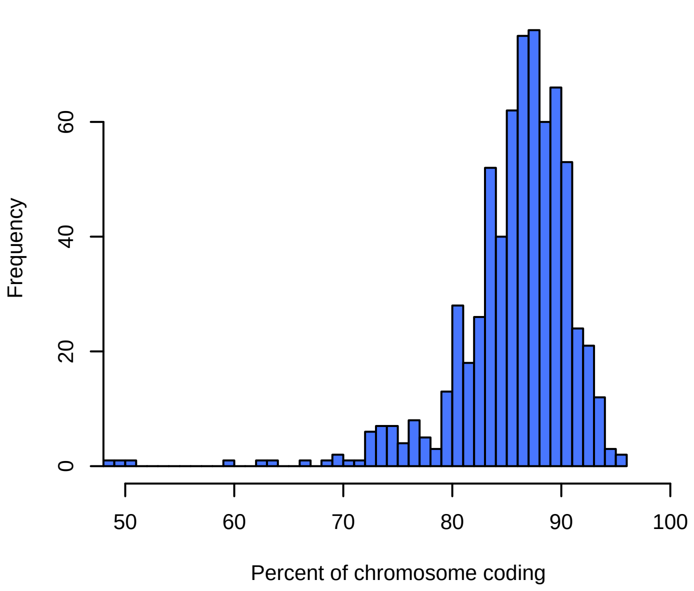

Coding oligomers in Frame 1

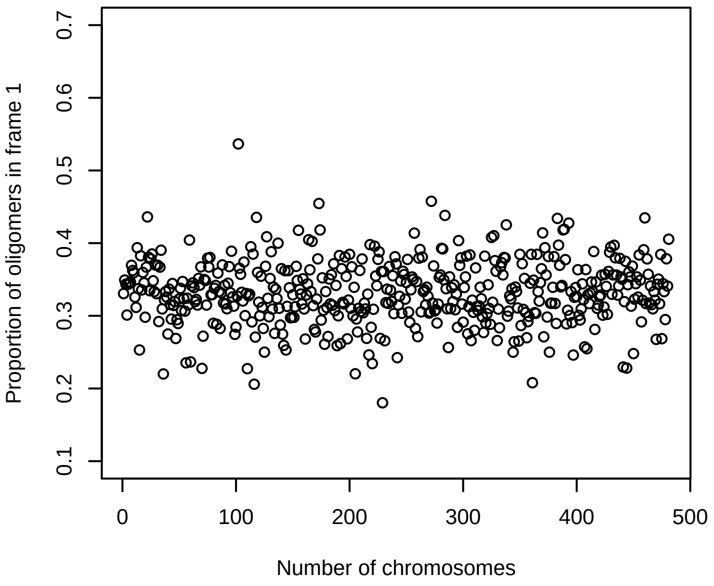

Coding oligomers in Frame 2

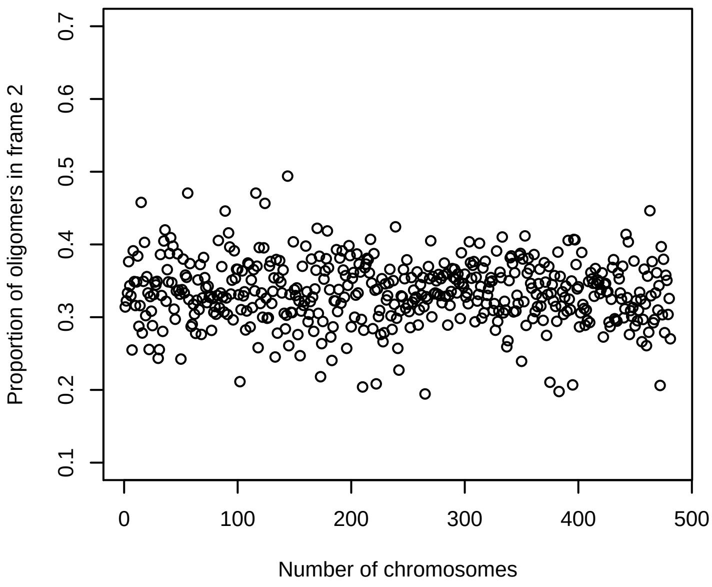

Coding oligomers in Frame 3

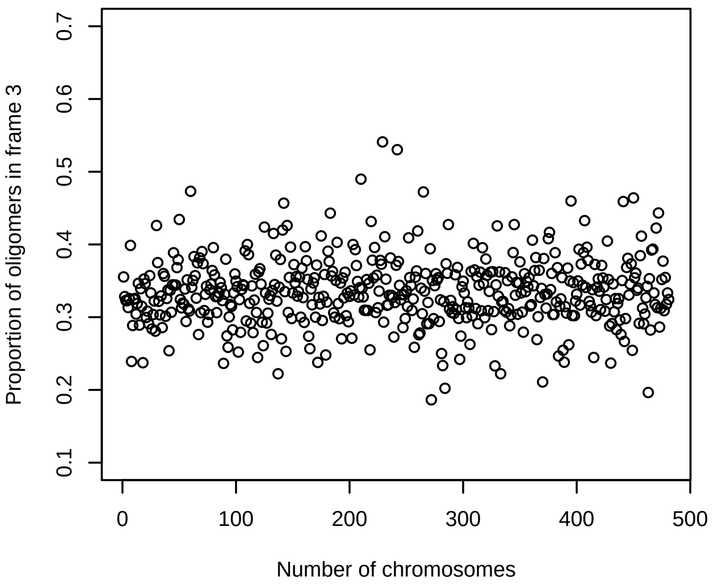

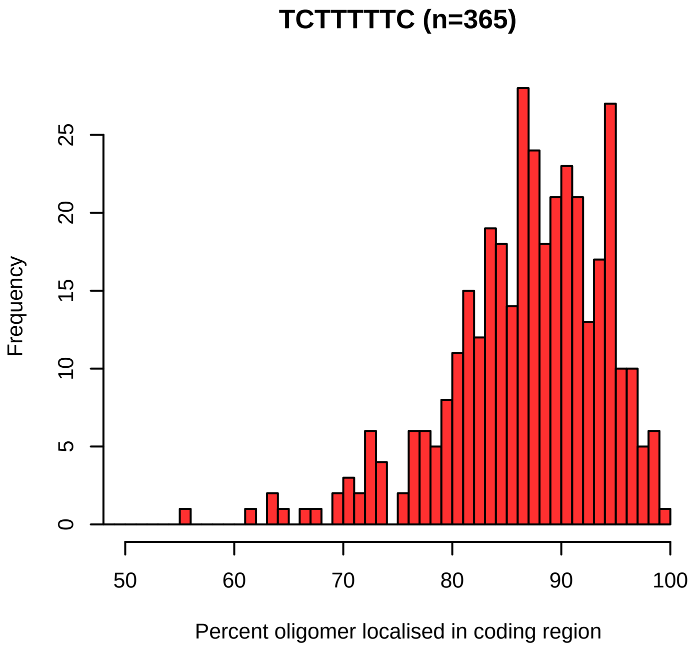
TCTTTTTC (n=365)
Frequency
Percent oligomer localised in coding region

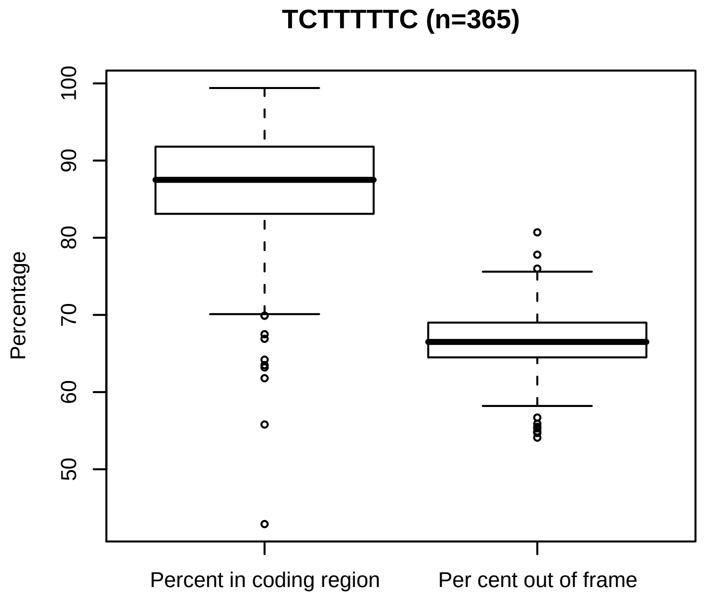
TCTTTTTC (n=365)
Percentage
Percent in coding region
Per cent out of frame

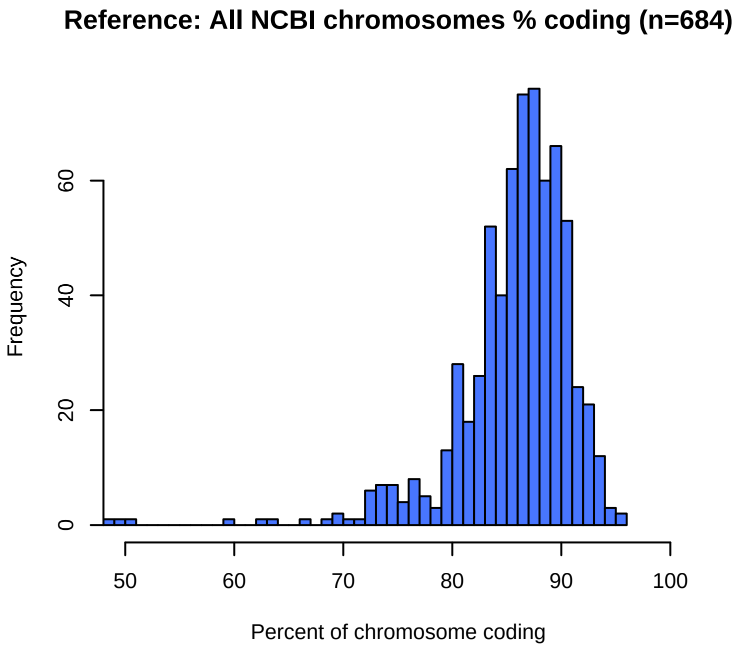
Reference: All NCBI chromosomes % coding (n=684)
Frequency
Percent of chromosome coding

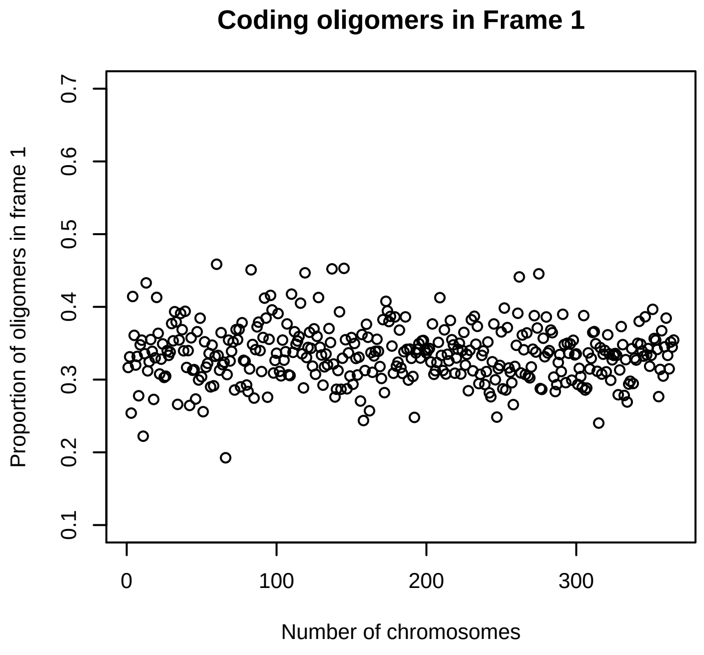
Coding oligomers in Frame 1
Proportion of oligomers in frame 1
Number of chromosomes

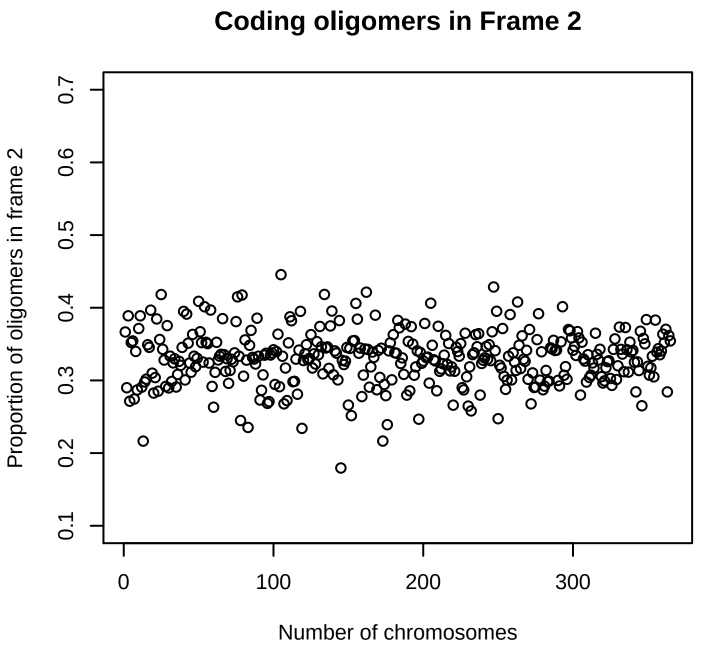
Coding oligomers in Frame 2
Proportion of oligomers in frame 2
Number of chromosomes

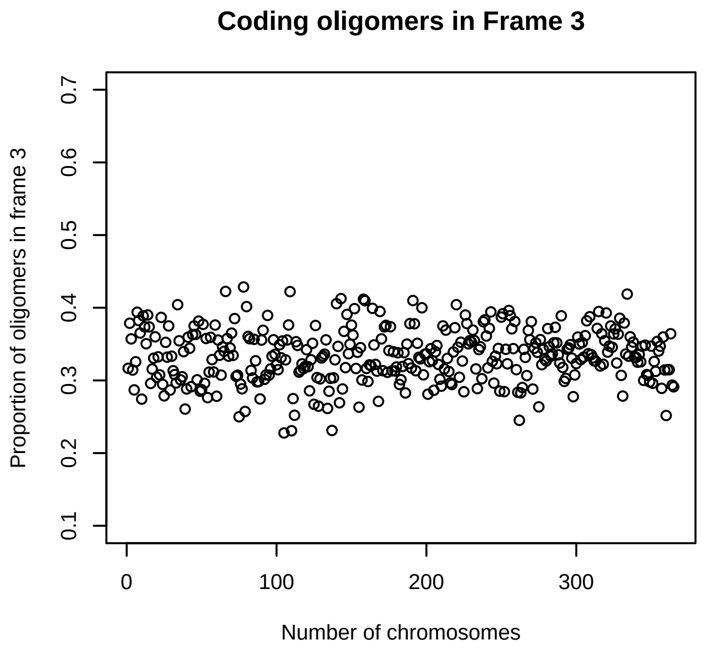
Coding oligomers in Frame 3
Proportion of oligomers in frame 3
Number of chromosomes

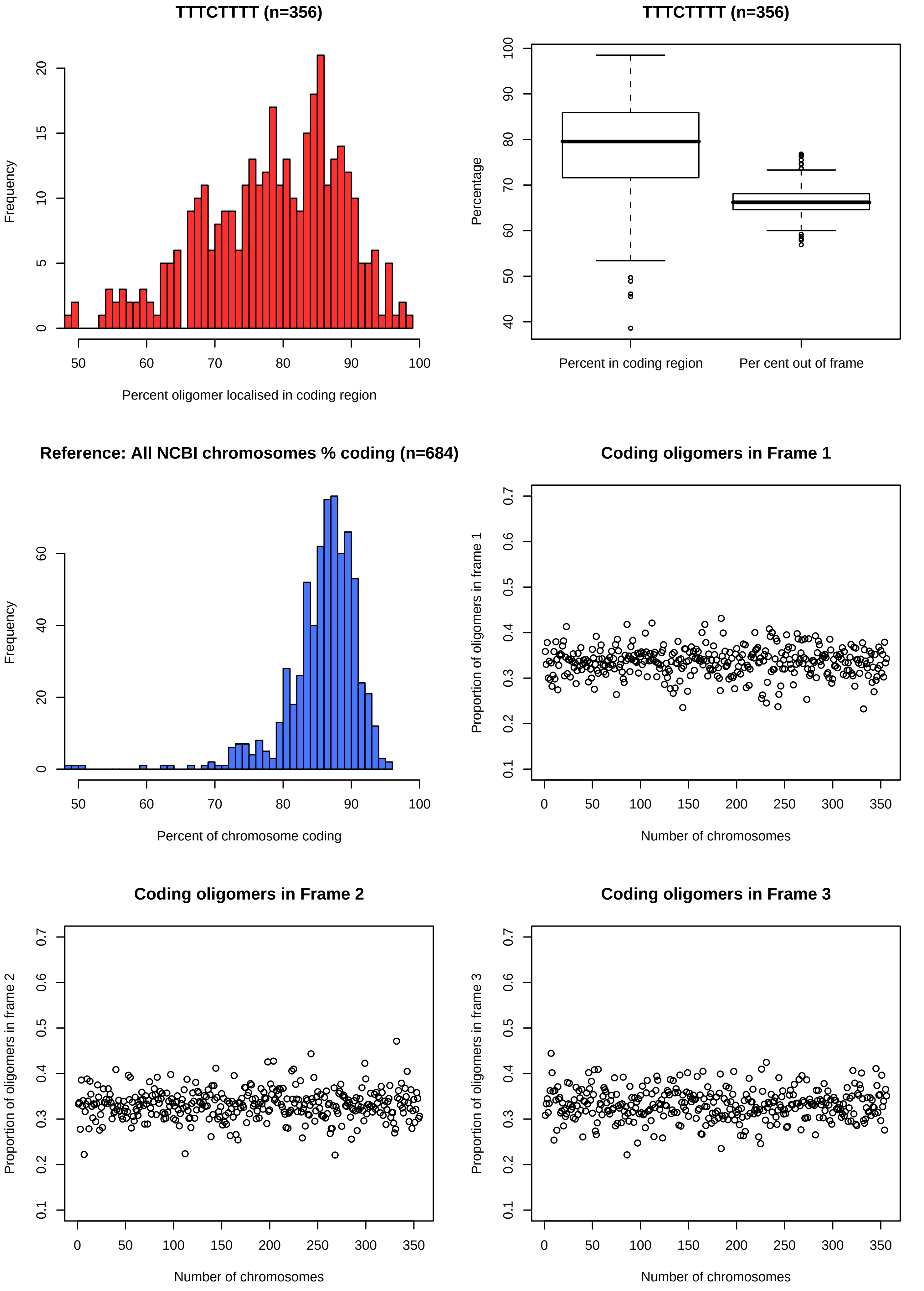
TTTCTTTT (n=356)
Frequency
Percent oligomer localised in coding region
TTTCTTTT (n=356)
Percentage
Percent in coding region
Per cent out of frame
Reference: All NCBI chromosomes % coding (n=684)
Frequency
Percent of chromosome coding
Coding oligomers in Frame 1
Proportion of oligomers in frame 1
Number of chromosomes
Coding oligomers in Frame 2
Proportion of oligomers in frame 2
Number of chromosomes
Coding oligomers in Frame 3
Proportion of oligomers in frame 3
Number of chromosomes

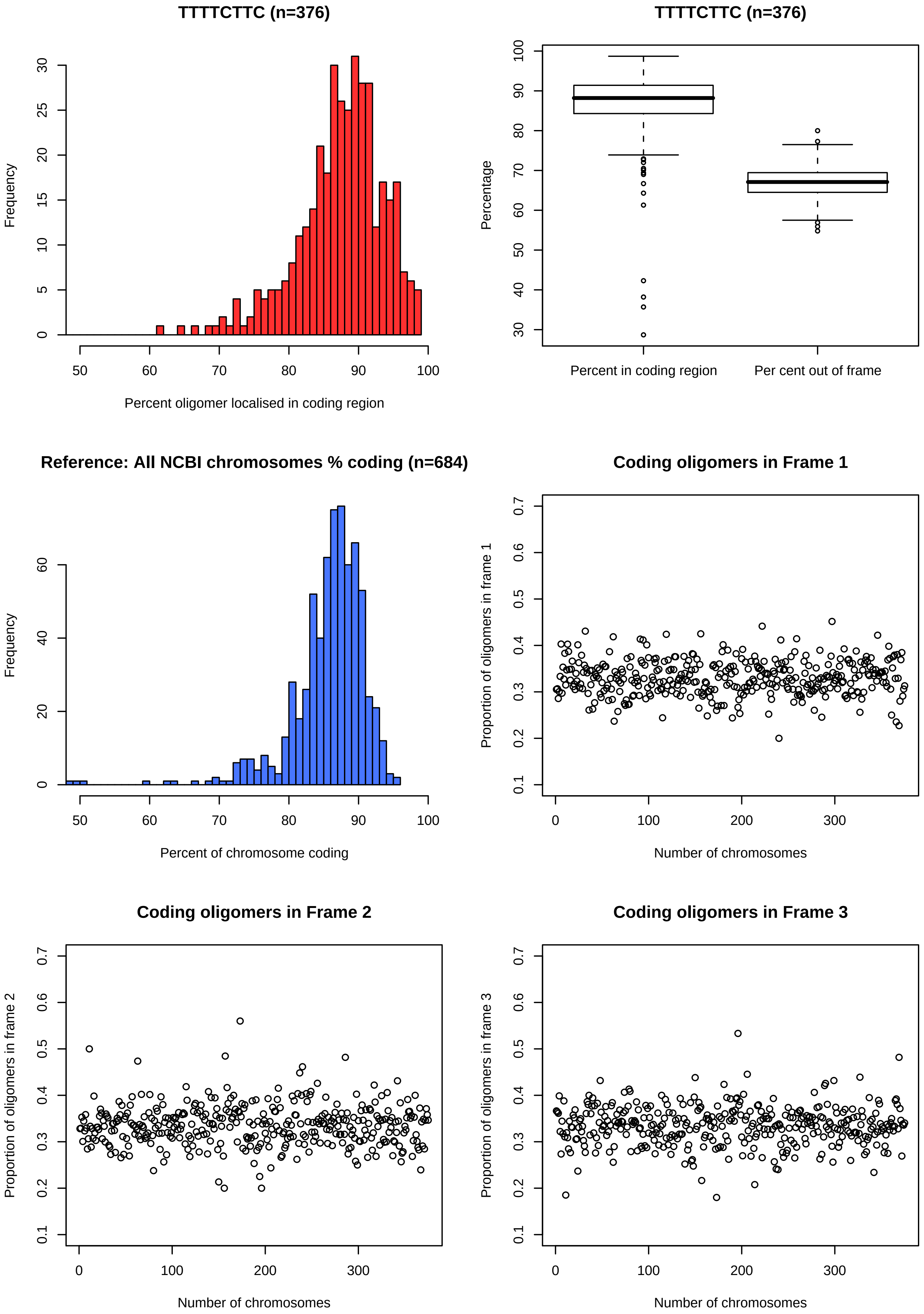
TTTTCTTC (n=376)
Frequency
Percent oligomer localised in coding region
TTTTCTTC (n=376)
Percentage
Percent in coding region
Per cent out of frame
Reference: All NCBI chromosomes % coding (n=684)
Frequency
Percent of chromosome coding
Coding oligomers in Frame 1
Proportion of oligomers in frame 1
Number of chromosomes
Coding oligomers in Frame 2
Proportion of oligomers in frame 2
Number of chromosomes
Coding oligomers in Frame 3
Proportion of oligomers in frame 3
Number of chromosomes

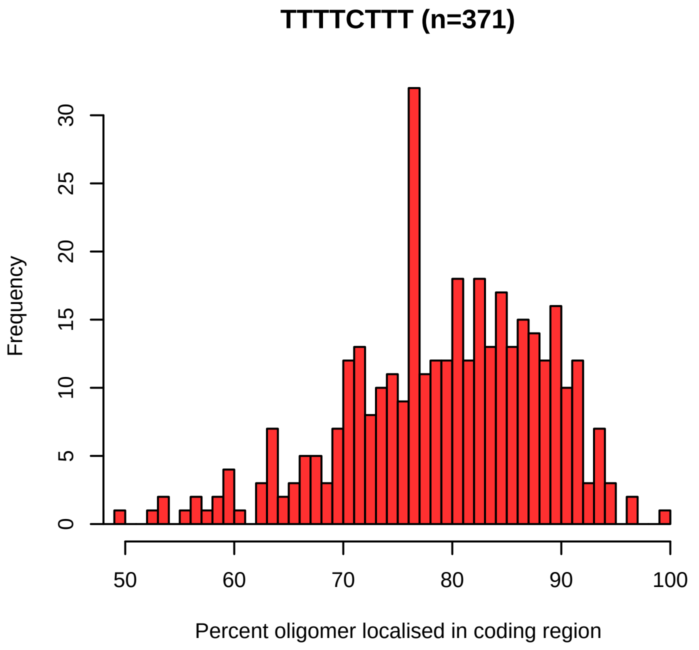
TTTTCTTT (n=371)
Frequency
Percent oligomer localised in coding region

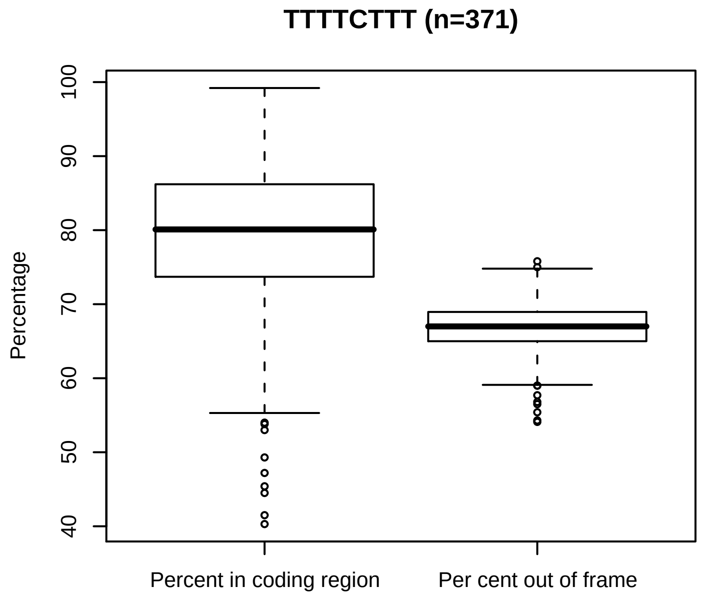
TTTTCTTT (n=371)
Percentage
Percent in coding region
Per cent out of frame

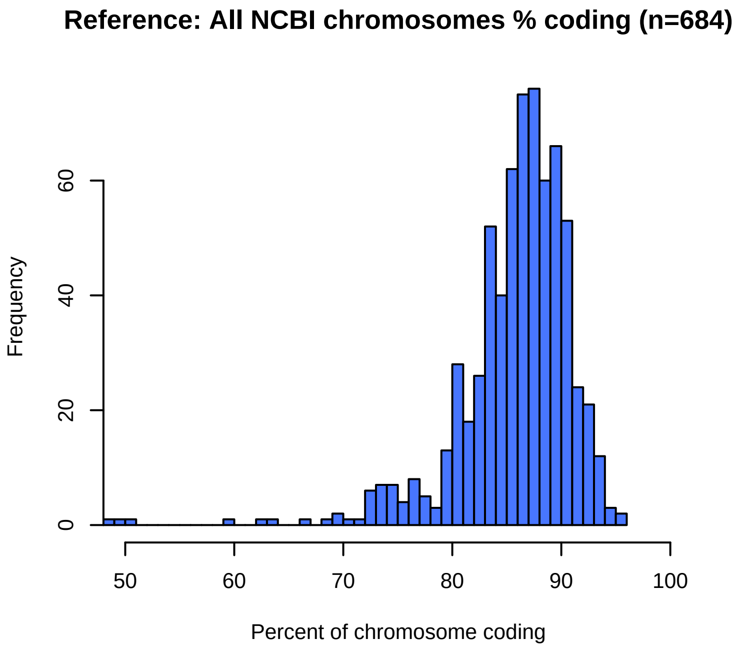
Reference: All NCBI chromosomes % coding (n=684)
Frequency
Percent of chromosome coding

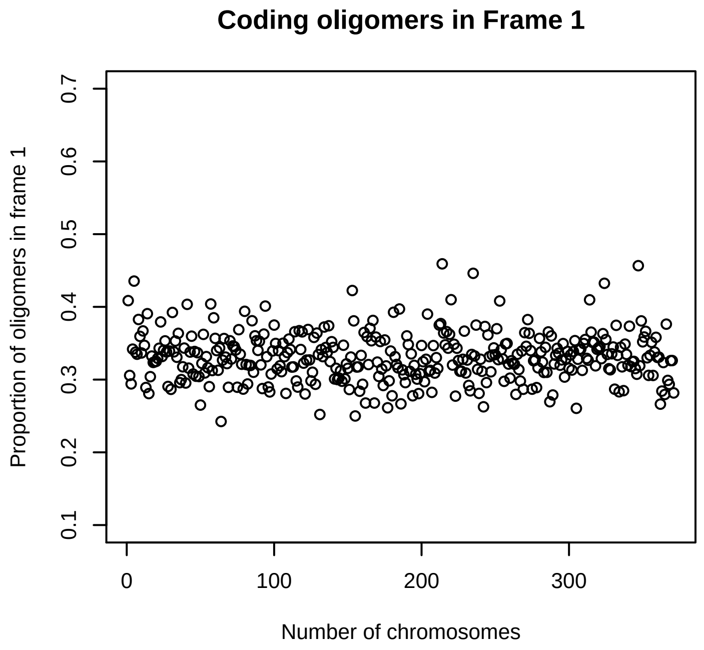
Coding oligomers in Frame 1
Proportion of oligomers in frame 1
Number of chromosomes

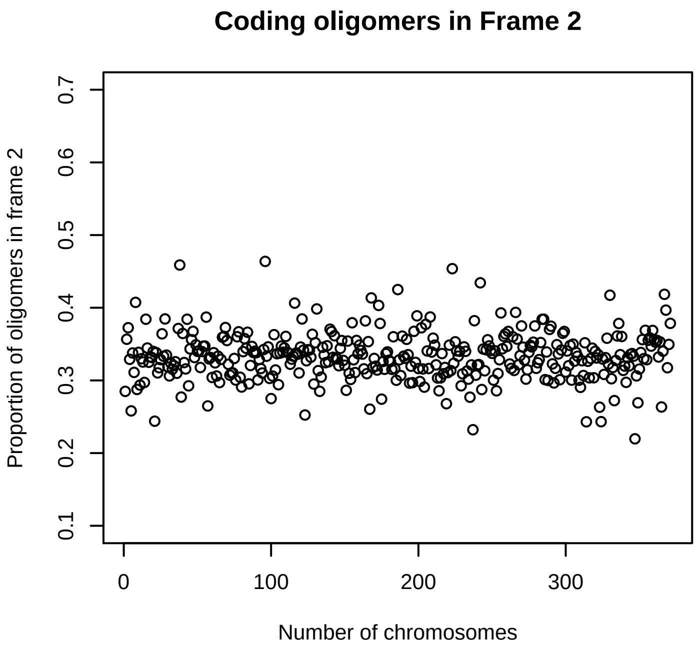
Coding oligomers in Frame 2
Proportion of oligomers in frame 2
Number of chromosomes

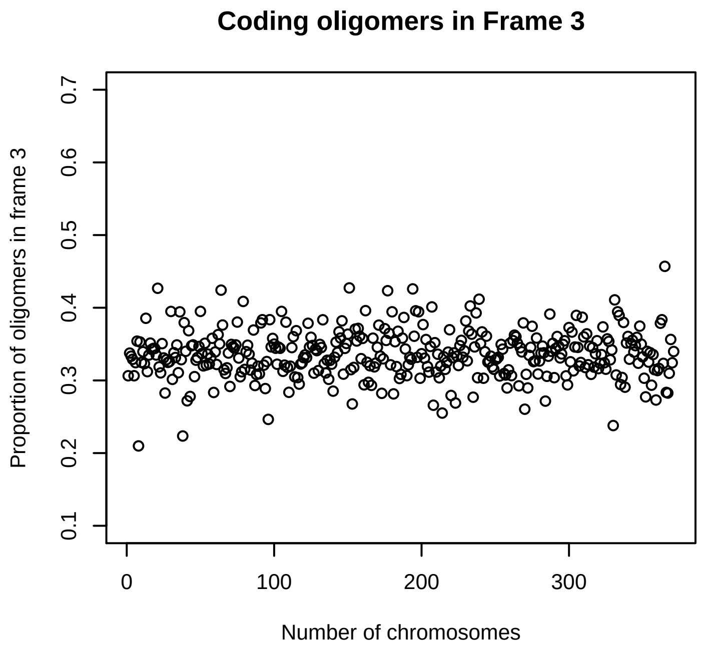
Coding oligomers in Frame 3
Proportion of oligomers in frame 3
Number of chromosomes

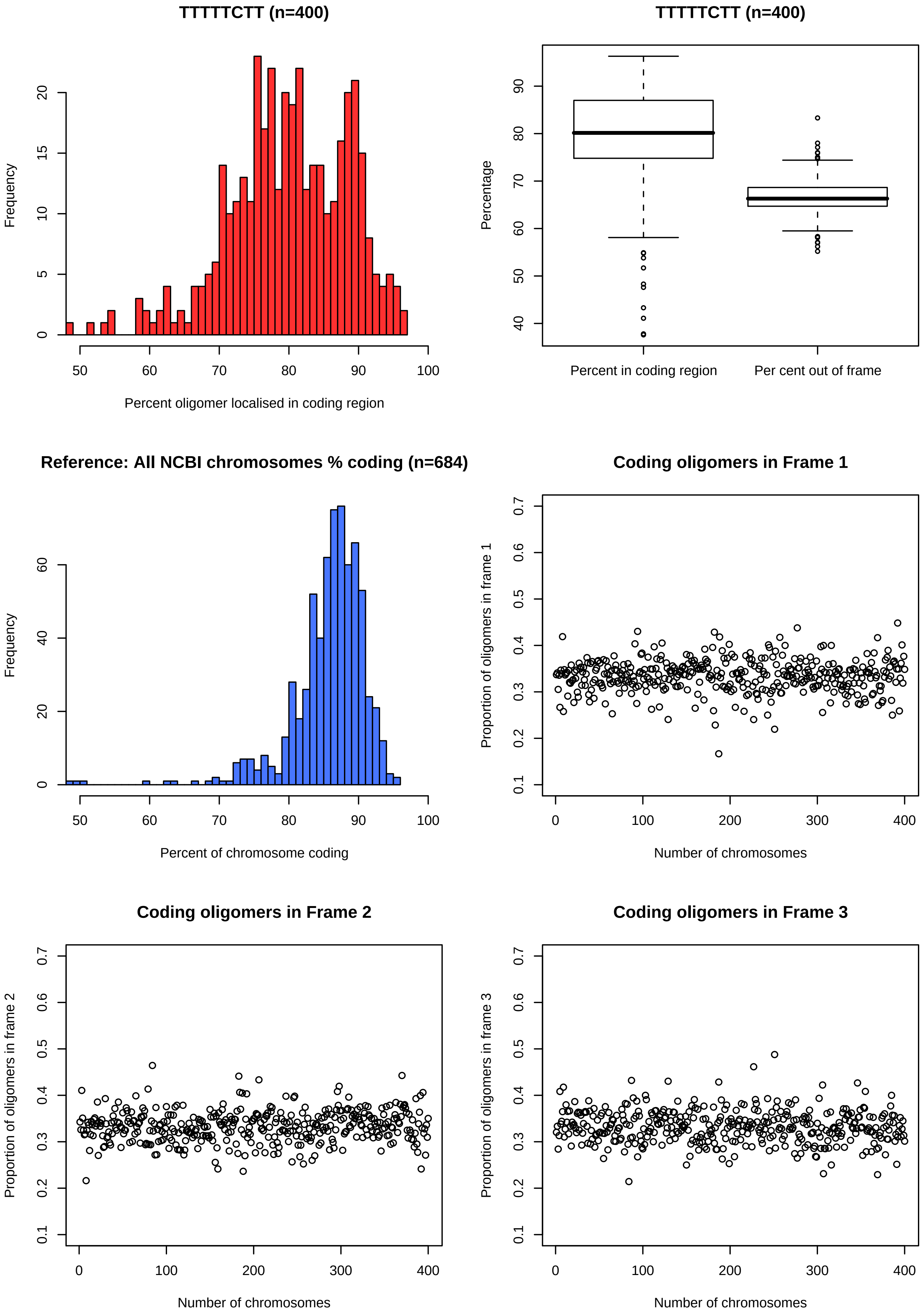
TTTTTCTT (n=400)
Frequency
Percent oligomer localised in coding region
TTTTTCTT (n=400)
Percentage
Percent in coding region
Per cent out of frame
Reference: All NCBI chromosomes % coding (n=684)
Frequency
Percent of chromosome coding
Coding oligomers in Frame 1
Proportion of oligomers in frame 1
Number of chromosomes
Coding oligomers in Frame 2
Proportion of oligomers in frame 2
Number of chromosomes
Coding oligomers in Frame 3
Proportion of oligomers in frame 3
Number of chromosomes
