# Supplementary material for: Abundant Oligonucleotides Common to Most Bacteria
Source: PLoS One. 2010 Mar 23;5(3):e9841. doi: 10.1371/journal.pone.0009841 (PMC2843746; doi:10.1371/journal.pone.0009841)
Supplement: Table S1 — Prokaryotic lineages lacking the top 15 overrepresented abundant oligomers. N indicates the number of genomes in the lineage. (0.29 MB PDF) [file pone.0009841.s002.pdf]

Supplementary Table S1. Prokaryotic lineages lacking the top 15 overrepresented abundant oligomers. N indicates the number of genomes in the lineage.

**Phylum**	**Oligo**	**Number of chromosomes oligo overrepresented in**
Acidobacteria	AAAGAAAA	0
Acidobacteria	AAGAAAAA	0
Acidobacteria	AGAAAAAG	0
Acidobacteria	CTTTTTCT	0
Acidobacteria	GAAAAAGA	0
Acidobacteria	TCTTTTTC	0
Acidobacteria	TTTCTTTT	0
Acidobacteria	TTTTCTTT	0
Acidobacteria	TTTTTCTT	0

(N=2; Candidatus *Koribacter versatilis* Ellin345, *Solibacter usitatus*)

Chloroflexi	CTTCTTCT	0

(N=6; *Chloroflexus aurantiacus* J-10-fl NC_010175, *Dehalococcoides* sp. BAV1 , NC_009455, *Dehalococcoides* sp CBDB1 NC_007356, *Dehalococcoides ethenogenes* 195 NC_002936, *Roseiflexus castenholzii* DSM 13941 NC_009767,*Roseiflexus* sp. RS-1 NC_009523)

Deinococcus-Thermus	AAAGAAAA	0
Deinococcus-Thermus	AAGAAAAA	0
Deinococcus-Thermus	CTTTTTCT	0
Deinococcus-Thermus	GAAGAAAA	0
Deinococcus-Thermus	TCTTTTTC	0
Deinococcus-Thermus	TTTCTTTT	0
Deinococcus-Thermus	TTTTCTTC	0
Deinococcus-Thermus	TTTTCTTT	0
Deinococcus-Thermus	TTTTTCTT	0

(N=5; *Deinococcus radiodurans* R1 NC_001263 and NC_001264, *Thermus thermophilus* HB8 NC_006461, *Deinococcus geothermalis* DSM11300 NC_008025, *Thermus thermophilus* HB27 NC_005835)

Epsilonproteobacteria	CCTTCTTC	0

(N=20; *Helicobacter acinonychis* Sheeba, NC_008229
Helicobacter hepaticus ATCC5144, NC_004917
Campylobacter curvus 525.92, NC_009715
Helicobacter pylori 26695, NC_000915
Campylobacter lari RM2100, NC_012039
Campylobacter jejuni NCTC11168, NC_002163
Helicobacter pylori J99, NC_000921
Sulfurovum sp. NBC37-1, NC_009663
Campylobacter jejuni subsp. *jejuni* 81-176, NC_008787
Campylobacter jejuni subsp. *doylei* 269.97, NC_009707
Nitratiruptor sp. SB155-2, NC_009662
Helicobacter pylori HPAG1, NC_008086
Campylobacter jejuni subsp. *jejuni* 81116, NC_009839
Campylobacter hominis ATCC BAA-381, NC_009714
Wolinella succinogenes DSM1740, NC_005090
Sulfurimonas denitrificans DSM1251, NC_007575
Arcobacter butzleri RM4018, NC_009850
Campylobacter jejuni RM1221, NC_003912
Campylobacter concisus 13826, NC_009802
Campylobacter fetus subsp. *fetus* 82-40, NC_008599)

Nanoarchaeota	CCTTCTTC	0

(N=1; *Nanoarchaeum equitans,* NC_005213)

Other bacteria	CTTCTTCT	0

(N=2; *Magnetococcus* sp. NC_008576, *Herminiimonas arsenicoxydans,* NC_009138)

Planctomycetes	AAAGAAAA	0
Planctomycetes	AAGAAAAA	0
Planctomycetes	AGAAAAAG	0

Planctomycetes	CTTCTTCT	0
Planctomycetes	CTTTTTCT	0
Planctomycetes	GAAAAAGA	0
Planctomycetes	GAAGAAAA	0
Planctomycetes	TCTTTTTC	0
Planctomycetes	TTTCTTTT	0
Planctomycetes	TTTTCTTC	0
Planctomycetes	TTTTCTTT	0
Planctomycetes	TTTTTCTT	0

(N=1; *Rhodopirellula baltica* SH 1)
